# Determining the relationship between mobile phone network signal strength and radiofrequency electromagnetic field exposure: protocol and pilot study to derive conversion functions

**DOI:** 10.12688/openreseurope.18285.2

**Published:** 2025-03-31

**Authors:** Nekane Sandoval-Diez, Lea Belácková, Adriana Fernandes Veludo, Hamed Jalilian, Florence Guida, Isabelle Deltour, Arno Thielens, Marco Zahner, Jürg Fröhlich, Anke Huss, Martin Röösli

**Affiliations:** 1 Epidemiology and Public Health, Swiss Tropical and Public Health Institute, Allschwil, 4123, Switzerland; 2 University of Basel, Basel, 4001, Switzerland; 3 Institute for Risk Assessment Sciences, Utrecht University, Utrecht, 3584, The Netherlands; 4 Environment and Lifestyle Epidemiology, International Agency for Research on Cancer, Lyon, 69007, France; 5 Ghent University, Ghent, Flanders, 9052, Belgium; 6 ASRC, The Graduate Center of the City University of New York, New York City, 10031, USA; 7 Fields at Work LLC, Zürich, 8032, Switzerland

**Keywords:** Radiofrequency electromagnetic field (RF-EMF) exposure, smartphone app, signal strength indicators, measurement protocol, spot measurements, conversion functions

## Abstract

Mobile phones continuously monitor and evaluate indicators of the received signal strengths from surrounding base stations to optimise wireless services. These signal strength indicators (SSIs) offer the potential for assessing radiofrequency electromagnetic field (RF-EMF) exposure on a population scale, as they can be related to exposure from both base stations and handset devices. Within the ETAIN (Exposure To electromAgnetic fields and plaNetary health) project, an open-access RF-EMF exposure app for smartphones, named "ETAIN 5G-Scientist”, has been developed using citizen science. This paper delineates a measurement protocol for deriving formulas to convert the app SSIs into electric field values to estimate RF-EMF exposure. It presents pilot study results from measurements taken at four locations in France (FR), and 14 locations in the Netherlands (NL), using three different phone models and the most common network providers in each country. The measurements were conducted while executing different usage scenarios, such as calls or data transmission. The exposimeter ExpoM-RF4 and on-body electric field probes were used to measure exposure from far-field sources and the handset, respectively. Two-minute aggregates were considered the sample unit for analyses (n=891 in NL, n=395 in FR). Regression analyses showed a positive log-linear relationship between Long Term Evolution (LTE) Received Signal Strength Indicator (RSSI) and far-field RF-EMF exposure when aggregating data by location (coefficients for normalised RSSI: 0.91 [95% CI: 0.55 - 1.28] in FR, 1.09 [95% CI: 0.96 - 1.22] in NL). Negative log-linear trends were observed for handset-related RF-EMF exposure at the ear (-0.31 [95% CI: -0.46 - -0.16]) and chest (-0.20 [95% CI: -0.37 - -0.03]) during data transmission scenarios. These results demonstrate that the ETAIN 5G-Scientist app can be implemented for smartphone-based RF-EMF estimation. However, uncertainties in individual measurement points highlight the need for further data collection and analysis to improve the accuracy of exposure estimates.

## Plain Language Summary

In this study, we looked at how mobile phones can help us measure exposure to radiofrequency electromagnetic fields (RF-EMF) from mobile wireless communication. As part of the ETAIN project (Exposure To electromAgnetic fields and plaNetary health), we developed an app called "5G Scientist" to keep track of the signal strength indicators (SSIs) that smartphones continuously use to establish and improve wireless connections. Our goal is to study how these indicators are related to RF-EMF exposure and to develop formulas for converting them into exposure values. To achieve this, we developed a measurement protocol and tested it in a pilot study. In this pilot study, we took spot measurements in France and the Netherlands using various phone models and network providers, while applying different usages of the phone, e.g. voice or video calls and data transmission. We used a personal exposimeter to measure exposure from far-field sources (such as base stations) and on-body electric field probes to measure exposure from near-field sources (such as own phone use). Our measurements indicate that as signal quality improves, exposure from mobile phone base stations increases, whereas exposure from one's own device decreases in both strength and duration. These results suggest that smartphone apps could serve as valuable tools for large-scale exposure assessment, as they show the potential to capture ambient exposure as well as exposure coming from the own mobile phone. However, more extensive measurement campaigns are needed to make this method more accurate.

## Introduction

To meet the ever-growing demand for enhanced connectivity in our modern era, wireless telecommunication technologies have consistently and rapidly evolved over the past few decades. The transition from the analogue networks of the first generation (1G) to the ultra-fast data speeds promised by the fifth-generation New Radio (5G-NR), has involved the introduction of new frequency bands and transmission techniques ^1^. The pervasive deployment of wireless communication infrastructures is accompanied by the escalating complexity and diversity of networks. As a result, exposure patterns may change, presenting new challenges in evaluating exposure to radiofrequency electromagnetic fields (RF-EMFs). Accurate exposure assessment is crucial for understanding and mitigating any public and scientific concerns regarding potential health effects associated with RF-EMF from wireless technologies.

Depending on the emitting source, two components of RF-EMF exposure can be distinguished. One is the uplink (UL) exposure, which is produced by the signals transmitted from the handset to the radio base station antennas. The other is the downlink (DL) exposure, which is composed of signals emitted by the base station antennas to user devices. Typically, UL exposure is self-induced, more localized, and originates from near-field sources situated within the Fraunhofer limit of the human body's position ^2^. In contrast, DL exposure has been considered environmental far-field exposure due to its association with fixed installations ^3^. With the advent of advanced technologies in 4G-LTE and 5G-NR base stations, such as beam-steering or Massive Multiple-Input Multiple-Output (MaMIMO), DL exposure dynamics have evolved ^4^. MaMIMO technology adjusts the phase and amplitude of the DL emissions to optimize the signal-to-noise ratio at the location of the receiving device, resulting in EMF beamforming ^5^. Consequently, DL exposure includes now a self-induced component that could result in increased RF-EMF exposure of users ^4, 6^.

Conventional RF-EMF exposure assessment approaches, reliant on fixed antenna patterns, face potential obsolescence with the adoption of adaptive antenna array systems. Additionally, the considerable spatiotemporal variability of RF-EMF continues to present challenges for accurate exposure assessment. The instruments used to record exposure quantities, comprehensively reviewed by Bhatt *et al.* ^7, 8^ are often expensive and can be bulky or heavy, making them cumbersome to carry. This limits measurements over extended periods of time or across large geographic areas and with large population samples. To circumvent these limitations, the use of smartphones as instruments to measure personal RF-EMF exposure has been proposed ^9– 11^.

Mobile phones are usually equipped with a multi-band antenna and routinely monitor the signal strengths of surrounding cells. Certain indicators of these signal strengths are calculated as means of quantification. These indicators are used in several network-related functions such as network discovery, connection establishment, and connection maintenance. Due to their role in connecting smartphones to the base stations and quantifying the received power at the connected antenna, signal strength indicators (SSIs) are potentially directly correlated with the DL component of the underlying electromagnetic field strength of the mobile radio network and, consequently, with ambient RF-EMF exposure ^12^. The Android operating system provides access to SSIs through its application programming interface (API) functions. However, information regarding the transmitted (UL) power of mobile phones during normal use is not readily accessible and can only be obtained from the network provider or by expanding the existing hardware of the phone. Understanding transmitted power is crucial for assessing the contribution of the UL to exposure ^12^. Despite this, SSIs indirectly impact the power transmitted by the handset by influencing its power control mechanisms ^13^. Several studies have reported a robust negative correlation between SSIs and transmitted power ^12, 14, 15^, due to path loss compensation and signal-to-noise ratio optimisation. When SSI is high, the phone is more likely to be closer to the base station, experiencing lower path loss and requiring less transmitted power to maintain a stable connection. Additionally, a stronger received signal improves the signal-to-noise ratio, allowing for more efficient communication. This negative correlation potentially allows for the utilisation of SSIs in estimating transmitted power, providing a framework for assessing both DL and UL RF-EMF exposure levels. Given the nearly universal use of smartphones, measurement data obtained through an app could, in principle, be used to estimate RF-EMF exposure on a population scale.

Currently, various smartphone applications offer the ability to collect information about SSIs from mobile and Wi-Fi networks. Examples include XMobiSense ^9^, ElectroSmart ^10^, Quanta Monitor ^16, 17^, and QualiPoc Android ^18^. Some of these apps have incorporated algorithms to estimate RF-EMF exposure based on the collected SSIs. ElectroSmart, for instance, provides an exposure index derived from Wi-Fi and mobile technologies. Quanta Monitor measures RF-EMF exposure (in μW m ^-2^) and offers real-time estimates of the specific absorption rate (SAR, in W kg ^-1^). However, there is limited scientific literature describing the methods and providing data used by these apps for converting app-generated data into power density, SAR or field strength values. Further, these relations may change over time in parallel with technological changes such as the introduction of 5G. Hence, it is difficult to rely on these apps for scientific studies.

Fröhlich *et al.* ^19^ and Schießl *et al.* ^20^ conducted feasibility studies to assess whether smartphones could be used to measure RF-EMF exposure. While they concluded that a fully comprehensive monitoring system is not feasible, they underscore that smartphone-based measurements can still provide valuable and reasonably reliable information. Similarly, Lopez-Espi *et al.* ^21^ proposed a methodology for exposure mapping using app-collected data and identified a proportional relationship between EMF measurements and SSIs. However, their approach lacked a conversion factor required to estimate exposure levels from smartphone data.

This study aims to investigate the relationship between SSIs and RF-EMF exposure related to mobile phone use, providing a reproducible framework for estimating RF-EMF exposure using an open-access smartphone app suitable for scientific studies. In this paper, we present the measurement procedures necessary for the derivation of conversion functions to estimate RF-EMF exposure from far-field sources and from the handset using cellular SSIs, along with results from a pilot study conducted to test the feasibility of the measurement protocol.

### Objectives

This work is part of the European Union’s Horizon-funded ETAIN (Exposure To electromAgnetic fIelds and plaNetary health) project. ETAIN takes a planetary health perspective to develop and validate approaches to assess the impact of existing and novel wireless technologies while exploring options for exposure reduction. Within this framework, we developed an open-access RF-EMF exposure app for smartphones, named "5G Scientist”, using a citizen science approach. To align with public expectations and needs, a co-design process involving citizens and experts determined the app's functionalities, interfaces, and relevant exposure indicators. The app is currently in the launch phase, and is already available on the Play Store ^22^. Data collected by European citizens will be used as input information to calculate spatially resolved exposure maps. These maps will offer insights into population exposure to RF-EMF from mobile telecommunication technologies.

This paper presents a measurement protocol that fulfils the following three key requirements ^19^: (1) recording RF-EMF exposure from all mobile network providers and various mobile radio technologies; (2) testing various common smartphone models; and (3) considering different positions of the smartphone in relation to users' bodies. In adherence to these requirements, the protocol was pre-tested in Lyon (FR) at four locations. Subsequently, it was applied as a pilot study in 14 locations in the Netherlands. The aim of the pre-test and pilot studies was to streamline the protocol for full implementation in two European countries, the Netherlands (NL) and Switzerland (CH), while also identifying key features for applying a shortened version in three other European countries: Germany (DE), Greece (GR), and Spain (ES).

## Methods

### Spot Measurement Protocol

***Measurement Locations and Study Areas***. Measurements are taken at different locations per country within two urban and two rural areas. Urban and rural areas are defined based on the level of urbanisation given by the harmonized definition of cities and rural areas of the European Commission ^23^. The number of locations per area comprises a minimum of five locations in urban areas and two locations in rural areas. The locations are selected based on the type of microenvironment, network density, and accessibility or ease of measurement. Initially, potential locations are identified according to microenvironment types as defined by the Corine Land Cover (CLC) nomenclature guidelines ^24^. This protocol focuses on four CLC classes considered relevant microenvironment types: city centre and residential areas (CLC codes: 111, 1121-1124), industrial and commercial areas (CLC code: 121), transportation network (1221-1223), and green areas (CLC codes: 141-142). To identify each type of microenvironment within each urban or rural area, the Urban Atlas 2018 ^25^ is used and vector layers for each area are displayed using GIS software ^26^. For urban areas, the allocation of locations per microenvironment is as follows: two locations in the city centre and residential areas (one indoor and one outdoor), one in industrial and commercial areas (indoor), one in transportation networks (indoor, usually the main train station), and one in a green urban area (outdoor). In rural areas, the distribution is one location in the town centre and residential areas (outdoor) and one in the transportation network (outdoor, usually local transport stations).

The final selection criteria to choose locations is based on the 4G-LTE and 5G-NR network density. National data on antenna locations is used for this purpose. In the context of this protocol, network density at a location is defined as the total number of deployed antennas within a one-kilometre circular area surrounding a location, encompassing coverage in the LTE-Advanced and 5G-NR era ^27^. Using GIS software ^26^, the network density is determined alongside the microenvironment type. Network density is used as a guide to select locations that represent different network load scenarios within each urban and rural area. Additionally, network density is used to select locations with comparable network loads across countries.

**Figure 1.**
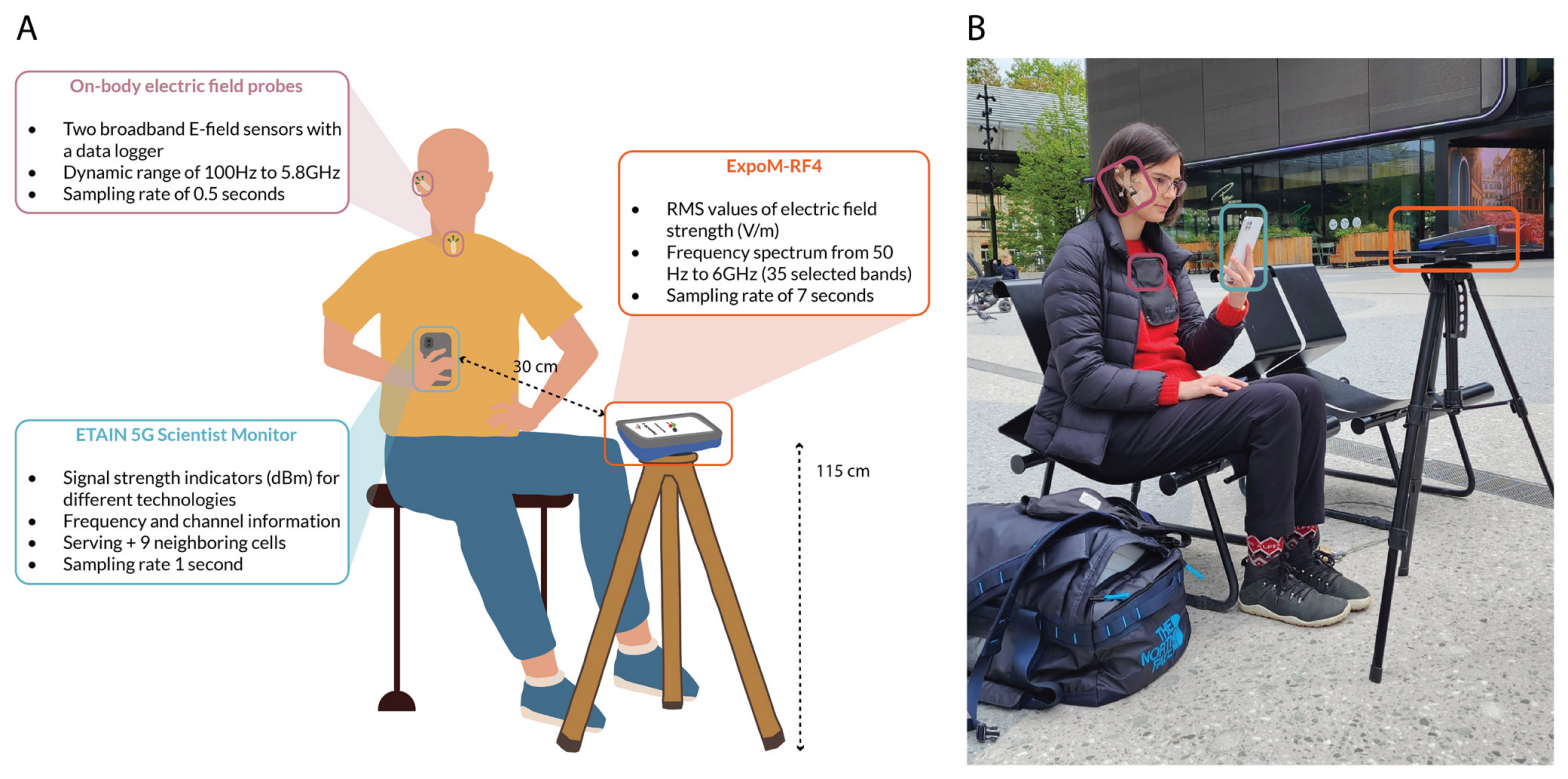
Illustration ( **A**) and real-life ( **B**) representation of spot measurement setup.

***Measurement Setup*.** At selected locations, measurements are carried out using a smartphone, on which the ETAIN 5G Scientist app is installed, a personal exposimeter (ExpoM-RF4 ^28^) to assess far-field exposure, and two custom-built electric field on-body probes ^29^ to evaluate exposure from the handset ( Figure 1). A trained researcher conducts simultaneous measurements with all tools while executing different usage scenarios with the phone (described below). The ExpoM-RF4 is mounted on a non-metallic tripod positioned in front of the researcher at a height of 115 cm and a minimum distance of 30 cm from the smartphone. The two on-body electric field probes are placed on top of the researcher's skin or clothes: one positioned in front of the tragus of the right ear and the other along the midline of the chest. The 5G Scientist app recordings are repeated using three different smartphone models supporting 5G-NR technology. To ensure access to representative data, the app measurements are also repeated using SIM cards from the most prevalent mobile network providers in each respective country (Table S1 in Extended Data ^30^).

***Measurement Tools***. The ETAIN 5G Scientist app uses data accessible through standard API functions and is currently implemented in the Android platform. It records data from the underlying operating system, providing information on cellular technologies and connected Wi-Fi and Bluetooth networks (Tables S2–S4, in Extended Data ^30^). The app logs the timestamp for each recording with local time in Coordinated Universal Time (UTC), with cellular data recorded every second and Wi-Fi/Bluetooth information recorded every ten seconds. Cellular information monitored by the app includes the most important SSIs for different technologies ( Table 1) ^31^, as well as frequency and/or channel information from the serving and up to nine neighbouring radio cells. SSIs are reported in dBm (decibel milliwatts: absolute power on a logarithmic scale), with varying ranges depending on the mobile communication standard. The serving cell, identified as index 0 in the app's recordings, refers to the specific cell within the network currently providing telecommunication services to the mobile phone. Neighbouring cells, indexed from 1 to 9 in the app's recordings, are adjacent to the serving cell and are part of the same network infrastructure of the subscribed provider. Additionally, the app tracks the total number of received and transmitted bytes over all networks. All information collected with the app is stored in the internal memory of the phone and can be downloaded as a CSV file.

**Table 1.** Overview of key Signal Strength Indicators * (SSIs) recorded with the ETAIN 5G Scientist app and their value ranges.

Technology	SSI	Description	Range [dBm]
3G-WCDMA	RSCP	Received power measured on one code of the pilot bits (reference signal) of a radio cell (usually the Primary Common Pilot Channel).	-120 to -25
4G-LTE	RSSI	Linear average of the total received power within the measurement bandwidth, including other power contributions besides the reference signal, such as neighbouring radio cells, interference, and thermal noise.	-113 to -51
	RSRP	Linear average of the power contributions of the resource elements that carry the cell-specific reference signal.	-156 to -44
5G-NR	SS-RSRP	Linear average of the power contributions of the resource elements that carry the secondary synchronization signal.	-156 to -31

*As defined by the 3rd Generation Partnership Project (3GPP) ^31^ SSI=Signal Strength Indicator; WCDMA= Wideband Code Division Multiple Access; LTE=Long Term Evolution; NR=New Radio; RSCP= Received Signal Code Power; RSSI= Received Signal Strength Indicator; RSRP=Reference Signal Received Power; SS-RSRP= Synchronization Signal Reference Signal Received Power.

The ExpoM-RF4, a portable frequency-selective personal exposimeter developed by Fields at Work GmbH ^28^, measures the Root Mean Square (RMS) and peak values of electric field strength (in V m ^-1^) across a frequency spectrum from 50 MHz to 6 GHz. We have established 35 frequency bands for measurement, encompassing the main frequencies used in mobile telecommunication technologies across Europe ^32^ (Table S5 in Extended Data ^30^). Sampling occurs continuously and is configured at an interval of seven seconds, during which every frequency band is measured for a duration of 50 ms. The upper limit of detection is 6 Vm ^-1^ for all frequency bands, while the lower limit varies from 0.0009 to 0.01 V m ^-1^ depending on the band.

The on-body field probes are novel wearable probe modules developed by Fields at Work GmbH with the aim of directly measuring RF-EMF near-field exposure on the body ^29^. Each probe consists of a flexible circuit board with three integrated broadband E-field sensors (channels A, B, and C) and a data logger (Figure S1 in Extended Data ^30^). These sensors use diode detectors to measure any EMF within a dynamic range of 100 to 5800 MHz at a sampling rate of two measurements per second. They feature a lower limit of detection of approximately 2 Vm ^-1^ and a saturation upper limit of up to 267 Vm ^-1^ (Table S6 in Extended Data ^30^). Each sensor produces a voltage signal proportional to the average electric field strength exposure, which is subsequently amplified, digitized, and stored in the data logger. The recorded voltage is then converted to field strength values based on a sensor-specific calibration ^29^.

***Usage Scenarios***. The measurements entail performing various scenarios with the phone, involving specific activities, telecommunication technologies, and positioning the handset relative to the body in a predetermined way. To ensure consistent measurements, we have identified 11 standardized usage scenarios ( Table 2). Each scenario involves one of six activities with a forced telecommunication technology. The activities include enabling flight mode, making a voice call using the phone's native services (i.e. without using any downloaded application), placing a voice call via a standard app such as WhatsApp, making a video call via WhatsApp, and continuously uploading a file to a File Transfer Protocol (FTP) server. Each activity is performed for two minutes. The phone's position relative to the body during the scenario is fixed for each activity, mimicking real-life situations. The operational technology of the smartphone is constrained within Android settings up to a certain technology (e.g. up to 4G or up to 5G) for the specific scenario. However, the actual technology used depends not only on the phone's settings but also on factors like network congestion, signal strength, and tower proximity. Thus, other legacy technologies may still be used throughout the scenario depending on these factors.

**Table 2.** Description of usage scenarios for spot measurements.

Scenario	Activity for 2 minutes	Technology *	Phone position
Non-use **	The phone is set in flight mode.	-	Held in the right hand in front of the body (~30 cm)
BT-music	Song playback using wireless earphones connected to the phone via Bluetooth.	Bluetooth	Held in the right hand in front of the body (~30 cm)
WiFi-WAvoice	Bidirectional reception and transmission of audio signals using WhatsApp.	Wi-Fi	Held in the right hand and pressed against the right ear
WiFi-WAvideo	Bidirectional reception and transmission of audio and video signals using WhatsApp.	Wi-Fi	Held in the right hand in front of the body (~30 cm)
WiFi-fileUpload	Continuous file upload to an FTP server.	Wi-Fi	Held in the right hand in front of the body (~30 cm)
4G-nativeCall **	Bidirectional reception and transmission of audio signals through the native service of the phone.	up to 4G-LTE	Held in the right hand and pressed against the right ear
4G-WAvoice **	Bidirectional reception and transmission of audio signals using WhatsApp.	up to 4G-LTE	Held in the right hand and pressed against the right ear
4G-WAvideo **	Bidirectional reception and transmission of audio and video signals using WhatsApp.	up to 4G-LTE	Held in the right hand in front of the body (~30 cm)
4G-fileUpload **	Continuous file upload to an FTP server.	up to 4G-LTE	Held in the right hand in front of the body (~30 cm)
5G-WAvideo **	Bidirectional reception and transmission of audio and video signals using WhatsApp.	up to 5G-NR	Held in the right hand in front of the body (~30 cm)
5G-fileUpload **	Continuous file upload to an FTP server.	up to 5G-NR	Held in the right hand in front of the body (~30 cm)

*Technology constrained within Android settings. **Usage scenarios conducted in the pre-test and pilot studies. BT=Bluetooth; WAvoice=WhatsApp voice call; WAvideo=WhatsApp video call; LTE=Long Term Evolution; NR=New Radio.

The implementation of the usage scenarios during measurements is facilitated through a bespoke mobile application called “ETAIN-scenarios”, which minimises the variability attributable to trained researchers and maximises protocol standardisation. Depending on the specific usage scenario, this app serves both as an instructional manual and, in some cases, enables certain actions during the measurements (Table S7 in Extended Data ^30^). Additionally, the ETAIN-scenarios app ensures that each scenario lasts two minutes, integrates error mitigation strategies and generates measurement timestamps that allow for temporal synchronisation between the recordings of all the measuring tools.

## Pre-test and pilot studies

Spot measurements were carried out in Lyon, France (FR), for a pre-test of the protocol, and in the Netherlands (NL) for a pilot study. In Lyon, four locations were chosen to represent various network load scenarios across the city. In the Netherlands, 14 locations were selected, encompassing two urban (Amsterdam and Utrecht) and two rural areas (Werkhoven and Zegveld) (Table S8 in Extended Data ^30^). The pilot study specifically focused on cellular technologies; thus, this paper details the measurements conducted for non-use, 4G, and 5G scenarios ( Table 2).

The measurement campaigns took place in FR from July 24 to 28, 2023, and in NL from October 3 to November 21, 2023. During these campaigns, data was collected using three different smartphone models and the most common network providers in each country. In Lyon, the phone models used were Samsung S22+, Xiaomi Redmi Note 11S, and Oppo A77, while the network providers included Orange, SFR, Free Mobile, and Bouygues Telecom. In the Netherlands, the same phone models were used as in France, except for a different Samsung model (Samsung A22), and the network providers were KPN, Odido, and Vodafone. All smartphones operated on Android version 13 (API level 33).

### Data analysis and modelling

Conversion functions are needed to express app SSIs as RF-EMF exposure values (i.e., electric field strength values). These functions need to consider variables that could affect the parameters recorded by the app and their correlation with DL and UL RF-EMF exposure. The general form of the conversion functions, as shown in Equation 1, relates RF-EMF exposure from far-field sources or the handset, measured with the ExpoM-RF4 and on-body electric field probes respectively, to the SSIs recorded by the ETAIN 5G Scientist app:

$$Y_{i}=\beta_{0}+\beta_{1}X_{i}+\varepsilon_{i}\;(Eq.1)$$

Where *Y _i_* is the response variable for the i-th observation (RF-EMF exposure in dBμVm ^-1^), *β*_0_ is the intercept (in dBm), *β*_1_ is the coefficient for the predictor variable *X _i_*, *X _i_* denotes the normalised SSIs (in dBm) at frequency *f*_0_, and *ε _i_* is the error term for the i-th observation.

According to the Friis transmission equation ^33^, when a transmitting antenna (e.g., a base station) emits a signal with certain power, and both transmitting and receiving antennas (e.g., phone) maintain constant gains, the received power is directly proportional to the square of the wavelength. Thus, as frequency increases (resulting in shorter wavelengths), received power decreases, assuming all other variables remain constant. To compensate for this relationship between received power and frequency, SSIs are normalised to a specific frequency ( *f*_0_ = 1800 *MHz,* as a middle frequency in the spectrum used for mobile networks), when the received frequency is known ( *f _i_*), using the following formula:


$$X_{i}=SSI_{Measured}+20log_{10}(f_{i}/f_{0})\;(Eq.2)$$

To estimate far-field exposure using SSIs, we undertook log-linear regression analyses across three levels to derive three types of far-field conversion functions, each with its own limitations and implications (see Table 3). To derive near-field conversion functions, we performed log-linear regression analyses between the exposure originating from the handset measured with the electric field probes at the ear ( *E _ear_*) or chest ( *E _chest_*) and the SSIs of the serving cell (s-SSI). Exposure from the handset is influenced not only by the transmitted power emitted by the phone but also by the exposure duration while using it. Thus, we normalised the on-body electric field probe measurements for file upload scenarios by data throughput (in megabytes per second, Mbps). In this way, s-SSI serves as a proxy of the phone's transmitted power. Given the minimal data throughput during voice call scenarios, no normalisation was conducted for these scenarios. As a validation step, we replicated the analysis using the UL exposure measured with the ExpoM-RF4.

**Table 3.** Description of the analysis levels to derive conversion functions for estimating far-field RF-EMF exposure from mobile communication technologies.

	Aim	Regression variables	Implications	Limitations
**Technical** **conversion** **function**	To determine the user´s DL exposure from the active band used for communication.	Response variable (Y _i_):	Reflects the underlying technical relationship between received power and RF-EMF exposure in the receiving band, yet it does not represent the user's total far-field exposure.	Ignores DL exposure from other base station antennas not used by the phone, including those from other providers and neighbouring cells of the same provider.
		DL or TDD exposure for the receiving band (E _DL-ActiveBand_)		
		Predictor variable (X _i_):		
		SSI of the serving cell (s-SSI: sLTE-RSSI, sLTE-RSRP, or sNR-ssRSRP)		
**App** **conversion** **function**	To determine total DL exposure using all available information from an individual ETAIN 5G Scientist app user.	Response variable (Y _i_):	It can be implemented in the ETAIN 5G Scientist app to provide users with a relevant exposure value and to study RF-EMF exposure on a population level.	Provides no information on DL exposure from the user's own provider.
		Total DL or TDD exposure for each technology *, i.e. 4G-LTE (E _DL-LTE_) or 5G- NR (E _NR_)		
		Predictor variable (X _i_):		
		Sum of the SSIs from serving and neighbouring cells (a-SSI: aLTE-RSSI, aLTE-RSRP, or aNR-ssRSRP)		
**Map** **conversion** **function**	To offer information on total DL exposure aggregating by location.	Response variable (Y _i_):	It uses the information from multiple users of the app to estimate DL exposure at a given location. Can be used for creating RF-EMF exposure maps by aggregating data across time and geographical areas from multiple users of the ETAIN 5G Scientist app.	Provides no temporal or instantaneous information on DL exposure.
		Mean DL or TDD exposure by location for each technology *, i.e. 4G-LTE (E _DL-LTE_) or 5G-NR (E _NR_)		
		Predictor variable (X _i_):		
		Sum of the arithmetic means of the SSIs from serving and neighbouring cells (a-SSI: aLTE-RSSI, aLTE-RSRP, and aNR-ssRSRP) for each provider at each location.		

*4G-LTE exposure is calculated as the sum of exposure across 700, 800, 900, 1800, 2100, and 2600 DL or TDD bands measured with the ExpoM-RF4; while 5G-NR exposure is calculated as the sum of exposure across 3500 TDD bands measured with the ExpoM-RF4. DL=downlink; TDD=time division duplexing; LTE=Long Term Evolution; NR=New Radio; SSI=signal strength indicator; RSSI= Received Signal Strength Indicator; RSRP=Reference Signal Received Power; SS-RSRP= Synchronization Signal Reference Signal Received Power.

Two-minute aggregates, corresponding to the duration of each usage scenario, were considered the sample unit for regression analyses in the technical and app conversion functions. ExpoM-RF4 measurements and 5G Scientist app recordings were temporally synchronized with each other and with the usage scenario timestamps from the ETAIN-scenarios app. Aggregates were computed for each unique combination of location, phone, provider, and scenario (n=795 in NL and n=325 in FR) by taking the arithmetic mean of the app SSIs, and of the recorded ExpoM-RF4 and probe electric field strengths during the interval. Specifically, the SSIs received power values were converted in the linear scale (mW), averaged, and back-transformed to the logarithmic scale (dBm) for analyses and graphical and tabular representation. The received power of the SSIs referred to the serving cell (s-SSI) or to the sum of serving and all neighbouring cells (a-SSI) for each aggregate. In cases where the serving cell changed during the two-minute intervals for 5G and 4G scenarios, we further subdivided the aggregates based on the serving cell, resulting in a total sample size of 891 in the Netherlands and 395 in France. In both countries, we observed a maximum of three different serving cells during the two-minute aggregates, with two or more serving cells in 68 (20.9%) of the aggregates in France and 87 (10.9%) in the Netherlands.

For the map conversion function, location aggregates were used as the sample unit for regression analyses (n=14 in NL, n=4 in FR). These aggregates were calculated by averaging the app SSIs for each provider at each location, considering both the serving and neighbouring cells (a-SSIs). The averages for all available providers were then summed by location to represent the total received power. Aggregation was performed in the linear scale (mW), and the values were subsequently back-transformed to the logarithmic scale (dBm) for the analyses. Additionally, the arithmetic mean per location of the recorded ExpoM-RF4 total DL and TDD exposure for each technology was calculated (see below).

Apart from the SSIs, the 5G Scientist app provided details about the operational frequencies of both serving and neighbouring cells. The frequency was determined using the LTE Evolved Universal Terrestrial Radio Access (E-UTRA) Absolute Radio Frequency Channel Number (EARFCN), NR Absolute Radio Frequency Channel Number (NRARFCN), or UTRA Absolute Radio Frequency Channel Number (UARFCN), depending on the technology employed by the serving cell (Table S9 in Extended Data ^30^). Using the serving cell frequency information, exposure measured by the ExpoM-RF4 was assigned to the corresponding DL, UL, and TDD active band ( *E*_*DL*– *ActiveBand*_, *E*_*UL*– *ActiveBand*_, and *E*_*TDD*– *ActiveBand*_).

Prior to any computations involving the ExpoM-RF4 data, a cross-talk correction was applied for frequency bands less than 100 MHz apart, following the methodology described by Eeftens *et al.* ^34^. The electric field strengths recorded by the ExpoM-RF4 were transformed into power flux density, measured in mWm ^-2^, to facilitate the calculation of the aggregates and determine total ( *E _T_*), downlink ( *E _DL_*), uplink ( *E _UL_*), and Time Division Duplex ( *E _TDD_*) exposures overall and for each technology (i.e. *E*_*DL*– *LTE*_, *E*_*UL*– *LTE*_, and *E _NR_*). *E _T_* was calculated as the power density sum of all 35 bands. *E _DL_* was computed as the sum of the following bands: 700 DL (centre frequency: 770.5 MHz), 800 DL (808.5 MHz), 900 DL (942.5 MHz), 1400 DL (1479.5 MHz), 1800 DL (1842.5 MHz), 2100 DL (2145 MHz), and 2600 DL (2657 MHz). *E _UL_* was calculated as the sum of 700 UL (718 MHz), 800 UL (847 MHz), 900 UL (897.5 MHz), 1800 UL (1747.5 MHz), 2100 UL (2535 MHz), and 2600 UL (2535 MHz) bands. *E _TDD_* was determined as the sum of all TDD bands, i.e. 700 (748 MHz), 2600 (2592.5 MHz), and 3500 (3475, 3605, and 3735 MHz). By technology, *E*_*DL*– *LTE*_ and *E*_*UL*– *LTE*_ represent the sum of power densities across 700, 800, 900, 1800, 2100, and 2600 DL, UL or TDD bands; whereas *E _NR_* is the sum of 3500 TDD bands. Upon completing the necessary computations, the results were converted to a logarithmic scale (dBμVm ^-1^) for modelling purposes as we expected a log-linear relationship between electric field strengths and the received power of the SSIs.

We performed a data quality assessment of the on-body electric field probe measurements. Measurements below the detection limit (<2 Vm ^-1^) were excluded from the analysis. In the Lyon dataset, the field probes encountered an electrical issue resulting in a loss of connection between the sensor and the data logger. Consequently, the field probe data collected in Lyon was deemed invalid and was excluded from the analyses. For the Netherlands dataset, the highest recorded value among the three channels of each probe was used as the proxy for peak exposure from the handset. Similar to the approach taken with the ExpoM-RF4 data, electric field strengths recorded by the probes were converted into power flux density (mWm ^-2^) for computing the arithmetic means of peak exposure over the two-minute aggregates. Since the electric field probes are not frequency-specific, we addressed environmental exposure by using measurements obtained during non-use scenarios specific to each combination of location, phone model, and network provider. For each aggregate, the exposure recorded during the corresponding non-use scenario was subtracted from the measured exposure during active usage. This subtraction process yielded adjusted exposure aggregates intended to isolate handset-related exposure. Subsequently, the reconverted electric fields were transformed onto a logarithmic scale (dBμVm ^-1^) for data analyses. As our focus is on exposure resulting from the phone's transmitted power emission, two-minute aggregates without successful data transmission (information provided by the 5G Scientist app) were not considered in the derivation of the near-field conversion function. From the data cleaning process described above, peak exposure at the ear occurred during voice call scenarios and file upload scenarios (n=85). Similarly, peak exposure at the chest occurred during the file upload scenarios (n=30). Regression analyses were conducted separately for scenarios where the phone was positioned against the ear (4G native and WhatsApp calls) and scenarios where the phone was in front of the body (file upload), as the distance between the phone and the electric field probe directly influenced the measured exposure.

All analyses were performed using R (version 4.3.2) within RStudio (version 2023.09.1.494). Kendall's tau was used to determine rank correlation coefficients, and linear regression was employed to establish the conversion functions.

## Results

### Descriptive statistics

**Figure 2.**
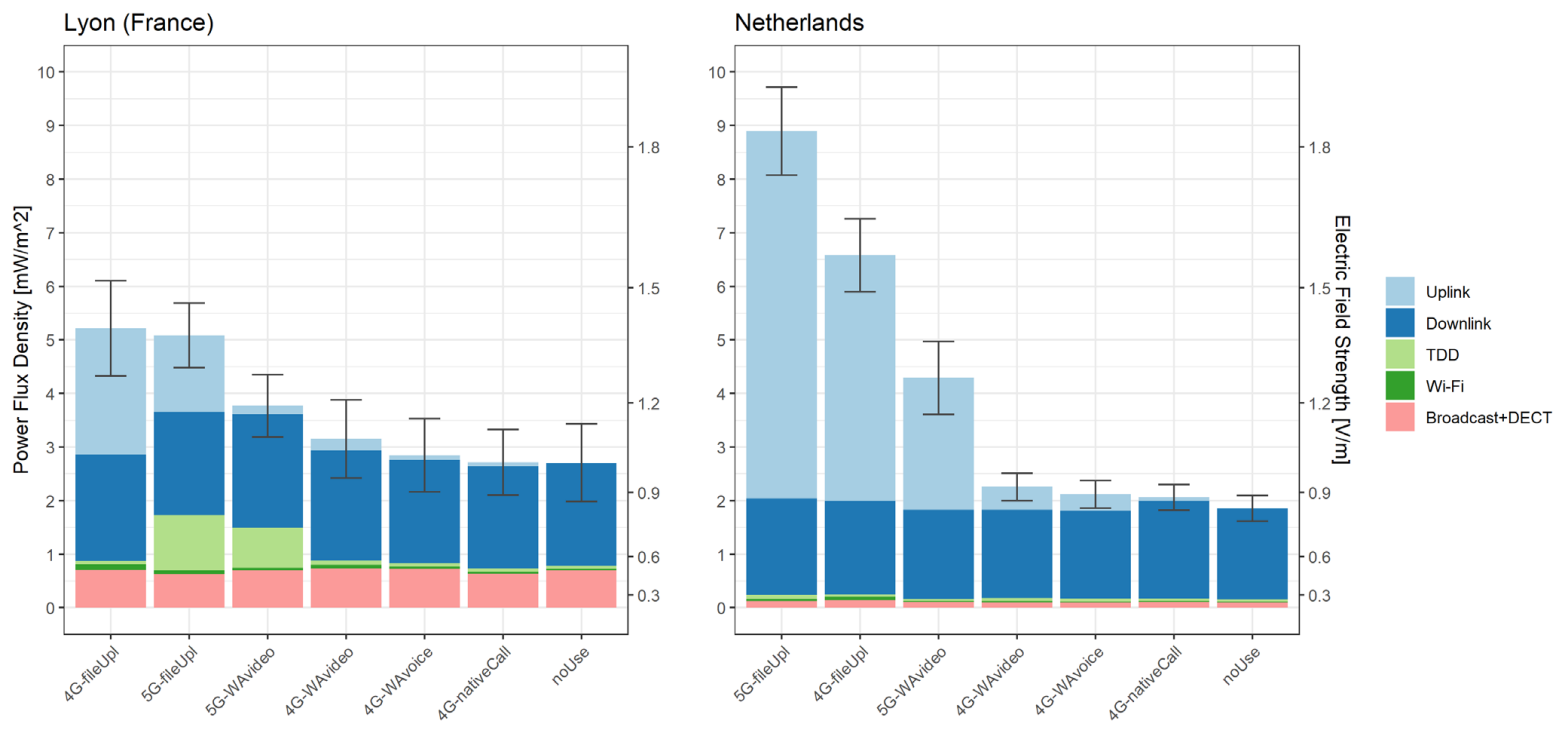
Mean exposure measured with the ExpoM-RF4 across all locations in each country by usage scenario. The colours indicate the contribution of each source category; the whiskers represent standard errors.

The mean total exposure across all locations during non-use scenarios was observed to be lower in NL compared to FR, with respective values of 0.84 Vm ^-1^ (Interquartile Range [IQR] =1.13 Vm ^-1^) and 1.01 Vm ^-1^ (IQR=2.40 Vm ^-1^). The difference in environmental exposure between the two countries was due to higher exposure levels in broadcast or infrastructure bands in Lyon (see Table S10 in Extended Data ^30^). Figure 2 shows the mean total exposure recorded by the ExpoM-RF4 per usage scenario in each country. Overall, there was virtually no difference in DL exposure between usage scenarios and it was similar for both countries (mean *E _DL_*: 0.86 Vm ^-1^ [IQR=0.26 Vm ^-1^] in FR and 0.80 Vm ^-1^ [IQR=0.55 Vm ^-1^] in NL). Variations in total exposure in the different scenarios were mainly due to differences in the UL and TDD contributions, which were the highest for the file upload scenarios. UL exposure was notably higher in NL in comparison to FR (mean *E _UL_* across all scenarios: 0.91 Vm ^-1^ [IQR=0.64 Vm ^-1^] in NL vs 0.49 Vm ^-1^ [IQR=0.38 Vm ^-1^] in FR), whereas TDD exposure was considerably higher in FR compared to NL (mean *E*_*TDD*_ across all scenarios: 0.38 Vm ^-1^ [IQR=0.30 Vm ^-1^] in FR vs 0.14 Vm ^-1^ [IQR=0.03 Vm ^-1^] in NL).

**Figure 3.**
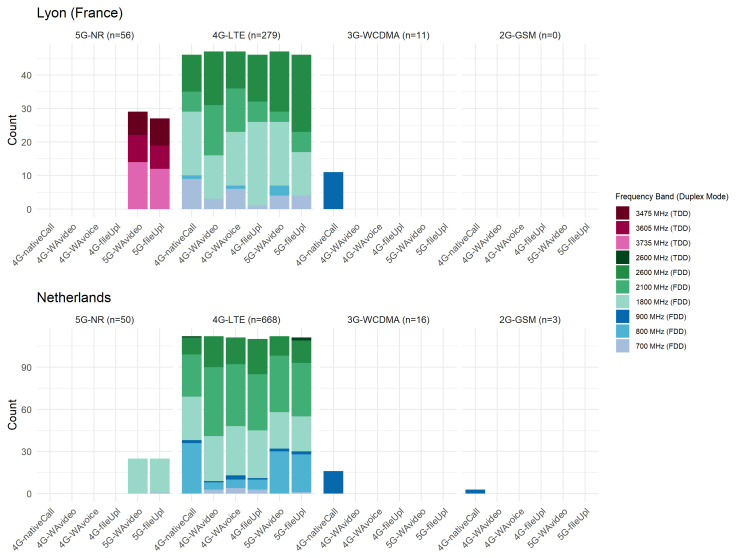
Distribution of operating frequency bands of the serving cell across usage scenarios and cellular technologies.

According to the 5G Scientist data ( Figure 3), 4G-LTE was the most commonly used technology by the serving cell, representing 80.6% of the aggregates in FR (n=279) and 90.6% in NL (n=668). 5G-NR was observed as the serving cell technology in 16.2% of aggregates in FR (n=56) and 6.8% in NL (n=50). Only a small percentage of serving cells, 3.2% (n=11) in FR and 2.6% (n=19) in NL, employed 3G-WCDMA or earlier legacy technologies, particularly during voice calls using native phone services (i.e. 4G-nativeCall usage scenario). Due to the limited sample size, no further analysis could be conducted using the WCDMA data. In scenarios where the operational technology was constrained up to 5G, the serving cell employed 5G-NR technology in 37.6% of cases in FR and 18.3% in NL, which was observed to be non-standalone in both countries. In NL, only one network provider (Vodafone) supplied 5G-NR technology; however, no 3.5 GHz bands were observed. Given the early phase of 5G-NR deployment in the Netherlands, 5G conversion functions were not analysed with the measurements conducted there. In both countries, a majority of operating frequency bands were observed in high-end frequencies, particularly of Frequency Division Duplex (FDD) type: 1800 MHz FDD (n=93 [26.9%] in FR, n=232 [31.5%] in NL), 2100 MHz FDD (n=49 [14.2%] in FR, n=241 [32.7%] in NL), 2600 MHz FDD (n=93 [26.9%] in FR, n=108 [14.7%] in NL). TDD and low-end FDD bands were observed less frequently ( Figure 3).

**Figure 4.**
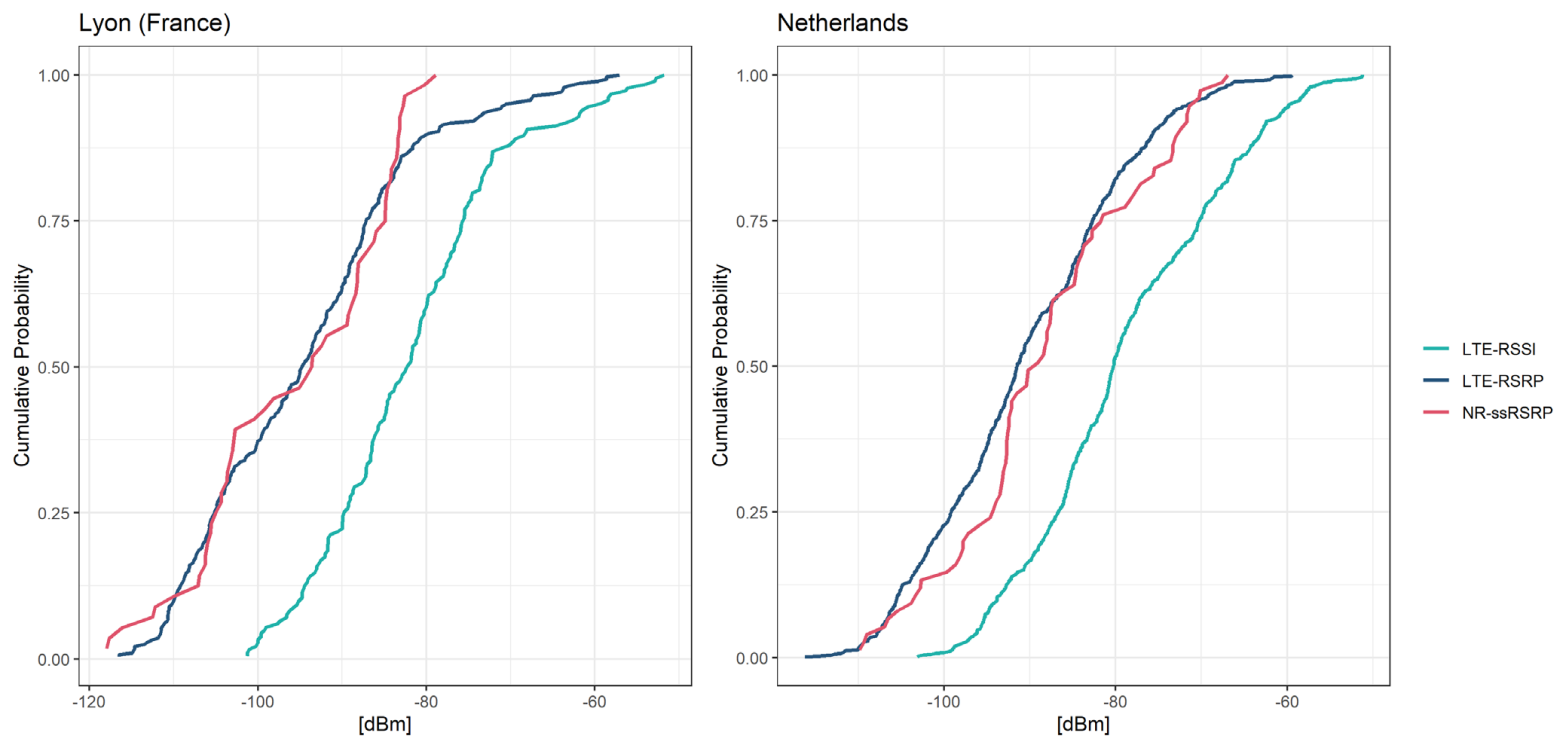
Cumulative distribution functions of the signal strength indicators measured with the 5G Scientist app.

In France, LTE-RSRP values varied between -116 to -57 dBm, with a median of -95 dBm, while LTE-RSSI values ranged from -101 to -52 dBm, with a median of -82 dBm ( Figure 4). Comparatively, in the Netherlands, LTE-RSRP values varied between -116 to -59 dBm, with a median of -91 dBm, while LTE-RSSI values ranged from -103 to -51 dBm, with a median of -80 dBm. NR-ssRSRP values were observed between -118 to -79 dBm in France and -110 to -67 dBm in the Netherlands, with median values of -94 and -89 dBm, respectively. The cumulative distribution functions (CDFs) of most SSIs were close to a Gaussian distribution ( Figure 4). Notably, in France, signal strength values above -70 dBm were less likely to occur compared to the Netherlands, indicating overall poorer signal quality in the Lyon selected locations. The CDFs did not indicate large variation between mobile phone models, but the distributions varied considerably between telecommunication providers (Figures S2–S5 in Extended Data ^30^). Additionally, when using the Samsung S22+ model in France, LTE-RSSI values were recorded for only 12 of the aggregates, but these values appeared implausible and were therefore excluded from further analysis. Furthermore, NR-ssRSRP values were not recorded at all using Samsung S22+ model in France. In all locations, LTE-RSRP and LTE-RSSI were found to be strongly positively correlated with each other (tau=0.89 with p-value <0.001 in FR, and tau=0.85 with p-value <0.001 in NL).

Table 4 displays summary statistics of the peak EMF exposure at the right ear for voice call and file upload scenarios, and provides a similar summary for peak exposure at the chest for file upload scenarios. On average, exposure at the ear during voice call scenarios exceeded that of file upload scenarios due to the direct placement of the phone against the ear. Exposure levels from the handset were higher at the chest than at the ear for both 5G and 4G file upload scenarios, with slightly higher levels observed for 4G compared to 5G. Additionally, data throughputs were higher in 5G compared to 4G file upload scenarios, with a median of 1.59 Mbps (IQR: 1.46) for 4G versus 1.75 Mbps (IQR: 2.04) for 5G ear exposure aggregates, and a median of 4.87 Mbps (IQR: 3.07) for 4G versus 5.31 Mbps (IQR: 3.95) for 5G in chest exposure aggregates.

**Table 4.** Summary statistics of peak EMF exposure at the ear and chest measured with the on-body electric field probes by usage scenario. Data from the Netherlands.

	Exposure from the handset measured at the right ear				Exposure from the handset measured at the chest	
	4G-nativeCall	4G-WAvoice	4G-fileUpl	5G-fileUpl	4G-fileUpl	5G-fileUpl
	[V m ^-1^]	[V m ^-1^]	[V m ^-1^]	[V m ^-1^]	[V m ^-1^]	[V m ^-1^]
**Min.**	0.81	1.06	1.42	1.63	2.06	2.03
**1 ^st^ Quartile**	2.58	3.12	1.90	1.81	2.49	2.33
**Median**	3.85	4.65	2.44	2.17	3.57	2.98
**Mean**	6.63	7.07	2.49	2.23	3.69	3.56
**3 ^rd^ Quartile**	8.41	7.88	2.80	2.58	4.25	3.75
**Max**	15.40	16.30	3.71	2.78	6.01	6.88
**IQR**	5.83	4.76	0.90	0.77	1.76	1.42
**N**	36	28	10	11	13	17

### Far-field conversion functions

**Figure 5.**
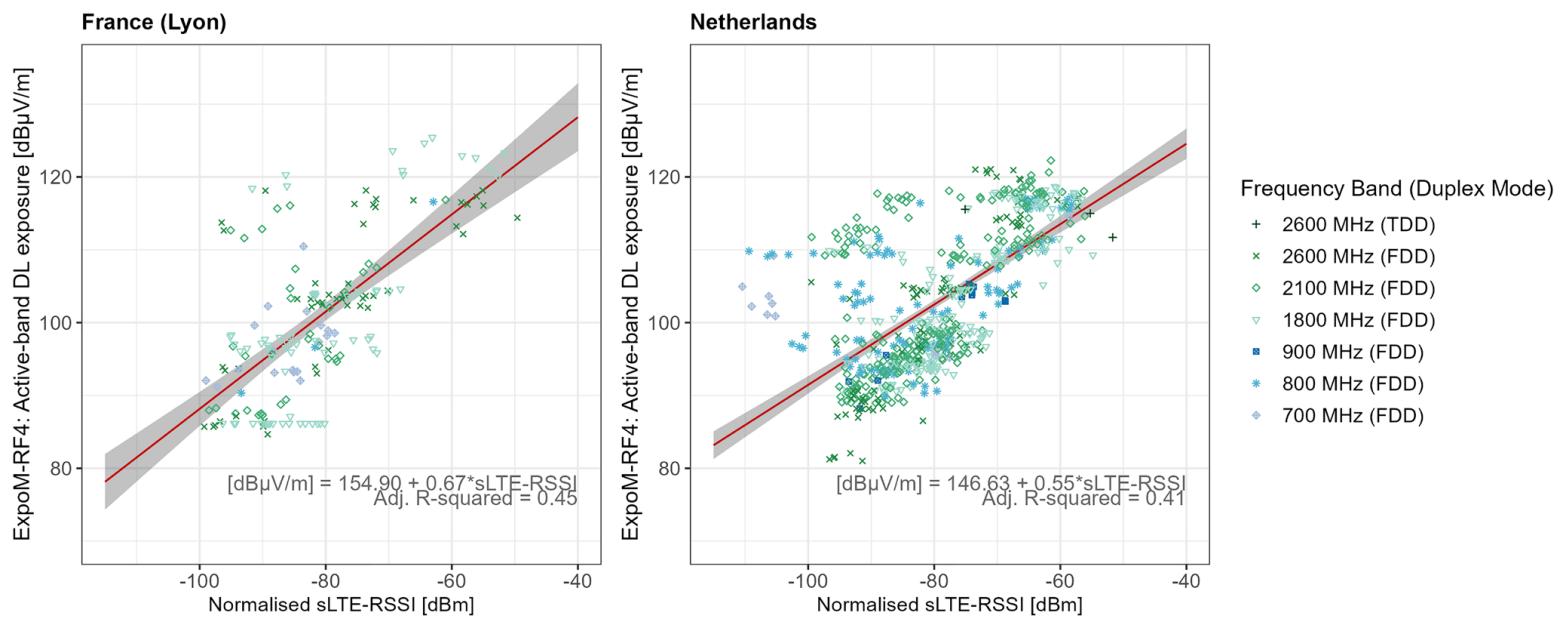
Regression analysis of active-band downlink field strength vs normalised LTE-RSSI from serving cell. The dots represent measurement aggregates (two-minute aggregates subdivided by registered cells) colour- and shape-coded according to frequency band. The red lines show the regression fit, and the grey areas represent 95% confidence bounds.

The regression estimates for the different far-field conversion functions are shown in Table 5. In the technical conversion models of both countries, sLTE-RSSI was found to be a significant predictor of DL active-band exposure (Likelihood Ratio Test (LRT) p-values <0.001), see Figure 5. The technical conversion models accounted for 41% (NL) and 45% (FR) of the variance in the data, with Root Mean Square Error (RMSE) of 0.28 for FR and 0.21 for NL. Additionally, Root Mean Square Logarithmic Error (RMSLE) values were 8.05 for FR and 7.42 for NL.

**Table 5.** Regression estimates for far-field conversion functions.

Far-field function	β _0_ * (95% CI)	β _1_ ** (95% CI)
Technical conversion		
France	154.90 (145.99 - 163.82)	0.67 (0.56 - 0.78)
Netherlands	146.63 (142.57 - 150.70)	0.55 (0.50 - 0.60)
App conversion		
France	158.61 (150.14 - 167.08)	0.63 (0.53 - 0.74)
Netherlands	150.11 (146.20 - 154.03)	0.51 (0.46 - 0.56)
Map conversion		
France	171.72 (146.70 - 196.74)	0.91 (0.55 - 1.28)
Netherlands	185.30 (176.38 - 194.22)	1.09 (0.96 - 1.22)

*Estimated intercept (in dBm) from log-linear regression analyses. **Estimated coefficient for the normalised LTE-RSSI value (in dBm) from log-linear regression analyses. 95% CI=95% confidence intervals.

**Figure 6.**
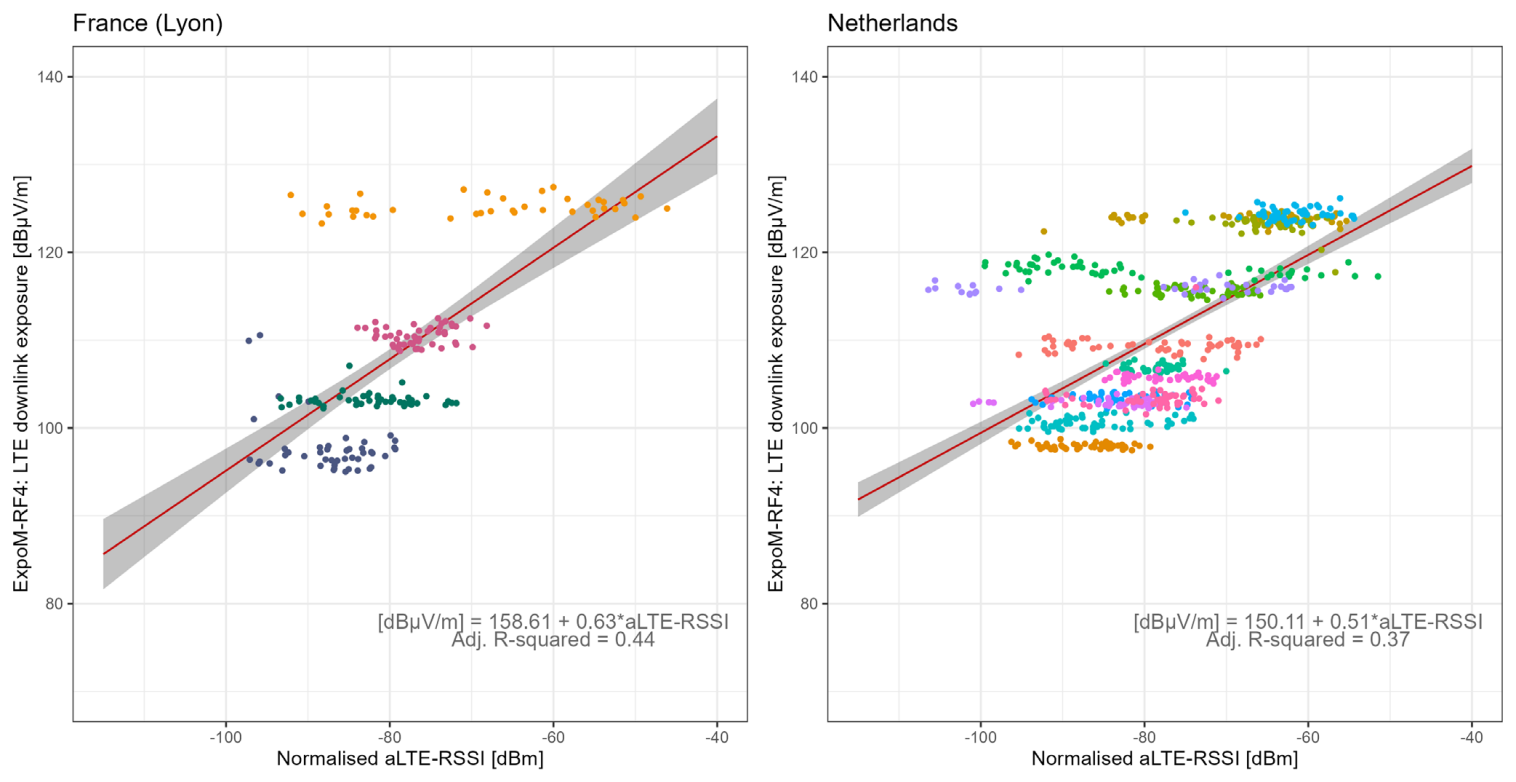
Regression analysis of LTE downlink field strength vs normalised LTE-RSSI from serving and neighbouring cells. The dots represent measurement aggregates (two-minute aggregates subdivided by registered cells) colour-coded by location, the red lines show the regression fit, and grey areas represent the 95% confidence bounds.

Figure 6 shows the relationship between all LTE-RSSI values of the own provider and 4G-LTE DL exposure from all providers. In the app conversion models, aLTE-RSSI was found to be log-linearly related to total DL exposure for 4G-LTE technology (LRT p-values <0.001). The fitted models explained 44% of the variance in FR and 37% in NL, with corresponding RMSE values of 0.55 and 0.45, and RMSLE values of 7.57 and 7.12, respectively. Compared to the technical conversion function, the app conversion models demonstrated lower performance in predicting DL exposure based on SSIs. Total DL exposure at a given location was influenced by base station density and proximity, reflecting geographic variations in cellular network infrastructure. Thus, DL exposure measured with the ExpoM-RF4 exhibited relatively low variability within a location, but high variability between locations ( Figure 6). In contrast, LTE-RSSI data exhibited substantial variability within each location ( Figure 6), since base stations from different providers are not evenly distributed across all areas and RSSI from a single network provider shows more variability over time than the aggregated exposure from multiple providers. Including an interaction term for the network provider improved the model performance (Figure S6 in Extended Data ^30^).

**Figure 7.**
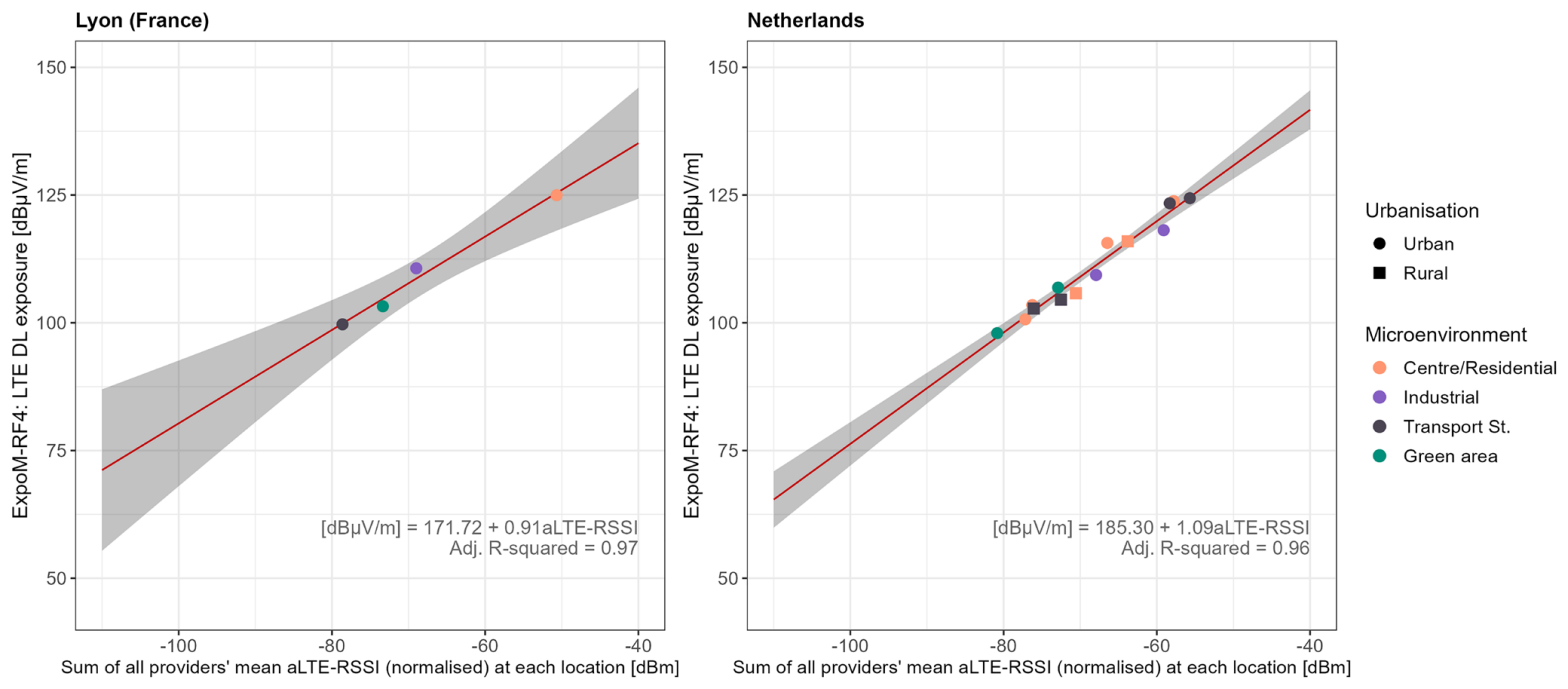
Regression analysis of mean LTE downlink field strength vs. providers' combined normalised aLTE-RSSI by location. The dots represent location aggregates, shape-coded by urbanisation and colour-coded by type of microenvironment. The red lines show the regression fit, while grey areas represent the 95% confidence bounds.

Figure 7 shows the correlation between the sum of the average LTE-RSSI values of all network providers and the average 4G-LTE DL exposure at each location. Aggregating data by location revealed that the cumulative mean aLTE-RSSI from all providers was a strong predictor of LTE downlink exposure (LRT p-values of 0.008 in FR and <0.001 in NL). For outdoor locations, aLTE-RSSI correlates positively with network density. The map conversion models explained over 95% of the variance in both countries, with RMSE values of 0.06 in FR and 0.14 in NL, and RMSLE values of 1.26 in FR and 1.64 in NL. Notably, the coefficients for aLTE-RSSI in both countries were remarkably similar in magnitude, adding to the robustness of the map function. The map conversion functions outperformed both technical and app functions, implying that while SSI values may serve as effective predictors of DL exposure on average, they may not accurately reflect instantaneous exposure levels.

Using LTE-RSRP instead of LTE-RSSI produced similar results for the technical, app, or map conversion functions (Figures S7–S9 in Extended Data ^30^). On average, coefficient estimates for LTE-RSRP were weaker and models typically exhibited worse performance than those using LTE-RSSI. Additionally, the results of the far-field conversion function for 5G-NR showed that NR-ssRSRP was not a significant predictor of neither active-band nor NR exposure, which was always from a TDD band (Figure S10 in Extended Data ^30^).

### Near-field conversion functions

**Figure 8.**
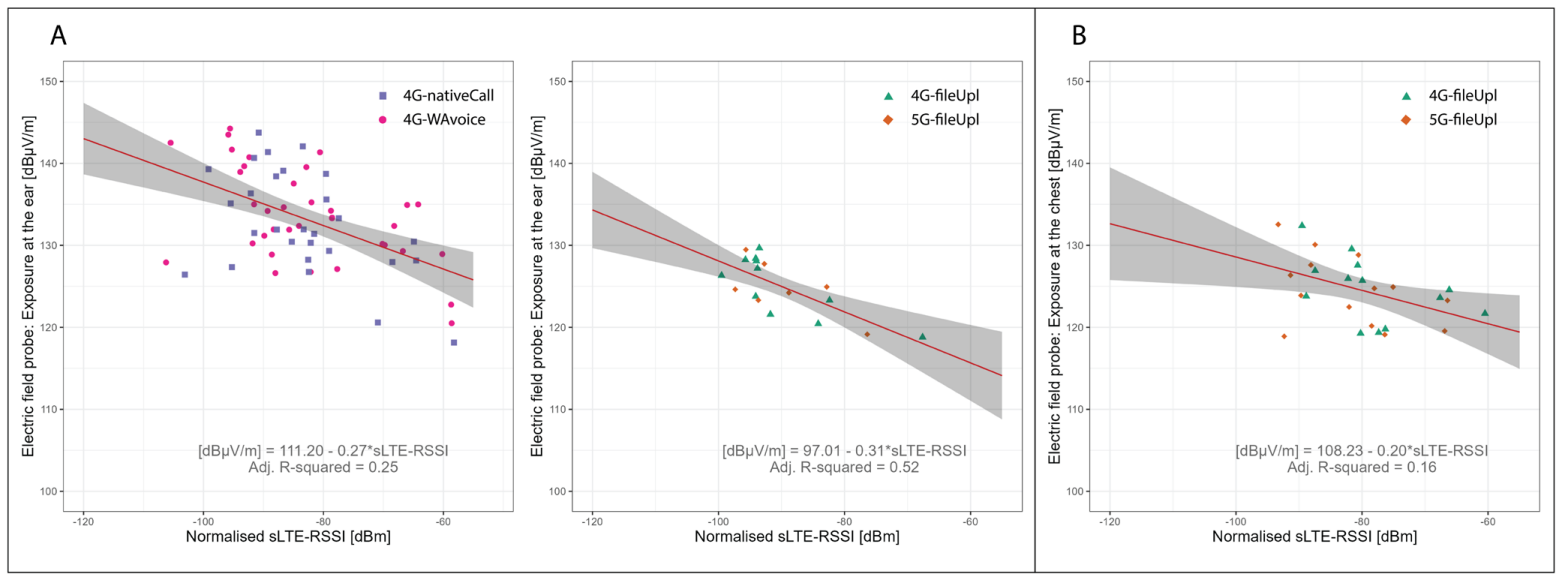
Regression analysis of handset-related EMF exposure at ear (A) and chest (B) vs LTE-RSSI. Regression analyses were stratified by phone positioning towards the ear (4G native and WhatsApp call scenarios) and in front of the body (file upload scenarios). The dots represent measurement aggregates (two-minute aggregates subdivided by registered cells) colour- and shape-coded by usage scenario. The red lines show the regression fit, while the grey areas represent the 95% confidence bounds. Data from the Netherlands.

Figure 8 illustrates the relationship between sLTE-RSSI and exposure from the smartphone at the ear and chest. The regression estimates for the near-field conversion functions at the ear and chest are shown in Table 6. LTE-RSSI from the serving cell exhibited a negative log-linear relation with ear exposure from the handset, either during voice call scenarios or file upload scenarios (LRT p-values <0.001). The relationship between sLTE-RSSI and chest exposure was weaker but displayed the same directional trend (LRT p-value of 0.020). The observed relationship between *E*_*UL*– *ActiveBand*_ measured with the ExpoM-RF4 and sLTE-RSSI also supports these findings (Figure S11 in Extended Data ^30^).

**Table 6.** Regression estimates for near-field conversion functions.

Near-field function	β _0_ * (95% CI)	β _1_ ** (95% CI)
Ear exposure		
Voice call scenarios	111.20 (101.81 – 120.60)	-0.27 (-0.37 - -0.15)
File upload scenarios	97.01 (83.52 – 110.50)	-0.31 (-0.46 - -0.16)
Chest exposure		
File upload scenarios	108.23 (94.64-121.82)	-0.20 (-0.37 - -0.03)

*Estimated intercept (in dBm) from log-linear regression analyses. **Estimated coefficient for the normalised LTE-RSSI value (in dBm) from log-linear regression analyses. 95% CI=95% confidence intervals.

## Discussion and study insights

This paper presents a measurement protocol designed to derive functions that convert signal strength indicators recorded with the ETAIN 5G Scientist app into RF-EMF exposure proxies. The protocol concept focuses on capturing the spectrum of dependencies in the assessment of the relationship between signal strength indicators and RF-EMF exposure from mobile phone use. Such dependencies include type of telecommunication technology, power ranges, network loads, phone models, network providers and usage patterns.

By establishing links between validated RF-EMF measurement methods and app-generated data, we were able to demonstrate that the 5G Scientist app can be implemented as a practical tool for collecting RF-EMF exposure proxies. Our findings reveal that aggregated SSI data from different network providers, strongly correlates with exposure from base station antennas, which is in line with feasibility studies conducted by Fröhlich *et al.* ^19^, Schießl *et al.* ^20^, and Lopez-Espi *et al.* ^21^. Among the SSIs, LTE-RSSI emerged as the most suitable proxy for far-field RF-EMF exposure from telecommunication technologies, as it encompasses a wide array of signal contributions, including both reference and interference signals ^30^.

Several sources of uncertainty must be acknowledged, as they may affect the confidence interval and validity of our conversion functions. In the technical conversion function ( Figure 5), one major source of uncertainty arises from the fact that the ExpoM-RF4 does not capture cell-specific signals but rather measures exposure from all contributing sources within each DL frequency band. This introduces variability, particularly at lower RSSI values, where measured exposure is more likely to be influenced by signals from other cells and users operating on the same frequency band. In contrast, at higher RSSI values, the measured exposure is more likely dominated by the serving cell’s signal, which is closer to what we want to predict using the technical calibration function. Similarly, a significant source of uncertainty in the app conversion function stems from relying on a single provider’s data to estimate total DL exposure ( Figure 6). At a given location, a user may experience poor signal quality from their own provider, resulting in low received power, while total DL exposure could still be high due to stronger power contributions from other providers’ base stations in the vicinity. This limitation introduces substantial uncertainty when estimating instantaneous exposure, which is why its implementation within the app was not pursued. Instead, we opted to provide an exposure estimate based on the technical conversion function, reflecting the user’s own cellular network. Incorporating data from multiple network providers, as demonstrated in the map conversion function ( Figure 7), improves the reliability of exposure estimates and addresses some limitations of both the technical and app conversion functions. Finally, while our methodology accounts for wavelength as a factor influencing the relationship between received power and measured RF-EMF exposure, it assumes constant antenna gains. In reality, antenna gains vary due to factors such as device orientation and the environment, introducing additional uncertainty in exposure estimation.

In Europe, the limited deployment of 5G-NR restricts our current conversion functions largely to 4G-LTE. Nevertheless, our study provides initial findings regarding the complex relationship between RF-EMF exposure and SSIs within the framework of 5G-NR technology. In TDD systems, where DL and UL transmissions occur simultaneously, the relationship between network quality and exposure becomes intricate. While better network quality may result in higher DL exposure due to stronger signal strength levels and closer proximity to base station antennas, it could conversely lead to reduced UL exposure. Better connectivity implies more efficient data transmission pathways with lower propagation loss and reduced interference, requiring less power from the mobile phone to transmit data. As the rollout of 5G-NR technology progresses across Europe, future measurement campaigns will provide additional insights into the relationship between SSIs and RF-EMF exposure in TDD systems.

We gathered data on EMF exposure at two body locations (ear and chest) during typical mobile phone use scenarios, observing different patterns of attenuation as EMF signals propagate from the handset to the user's body. For instance, exposure at the ear was approximately three times lower when the phone was held in front of the body compared to when it was in direct contact with the ear. Our results suggest that SSIs may serve as proxies for peak exposure from the handset. We found that exposure from one's own device decreases in both strength and duration with increased signal quality, aligning with the power control mechanisms of the phone ^12, 14, 35^. This result highlights the potential of SSIs as a useful metric for estimating handset-related EMF exposure.

Data from the pre-test and pilot studies was used to develop far-field and near-field conversion functions, which are going to be integrated into the first public 5G Scientist app version. These functions aim to provide users with exposure indicators associated with mobile phone use. Employing smartphones for RF-EMF exposure estimation offers a range of practical advantages. Capitalizing on the widespread use of smartphones in the population allows the collection of extensive datasets from diverse individuals and large geographical areas. This enhances the feasibility of conducting large-scale studies and opens avenues for robust epidemiological research on potential health effects linked to RF-EMFs. Moreover, the use of smartphones in exposure assessment aligns with the principles of citizen science, empowering individuals to actively contribute to research initiatives. This engagement not only fosters a sense of public participation but also enhances the transparency and inclusivity of scientific investigations.

### Protocol changes

The feasibility of the measurement protocol was successfully demonstrated in the pre-test and pilot studies, providing insights into the conversion functions and identifying areas for improvement in future measurement campaigns. Our data was somewhat scarce at the lower end of the received signal strength range (i.e., more negative values) where signal quality deteriorates. Capturing the full range of signal strength values is particularly important for the near-field conversion function, as power control mechanisms should reflect a capped maximum exposure with decreased signal strength beyond a certain threshold ^36^. In our pre-test and pilot studies, power control mechanisms for weak or very weak signal strengths were not fully achieved. Furthermore, more information is needed for low-end frequency bands (20% or less of the data recorded with the app during the pilot study corresponded to frequency bands of 900 MHz or lower). Therefore, to assess the full range of signal strength values in each study area, we have opted to expand measurements in indoor settings and add mobile measurements. For indoor locations selected in urban areas, additional measurements are going to be conducted at different indoor levels, including rooftops and basements.

Mobile measurements will be conducted in both urban and rural areas. In urban and rural settings, polygonal measurement zones, ranging from 0.5 to 1 km ^2^ each, will be defined. Trained researchers will simultaneously take recordings using the 5G Scientist app with the smartphones set to idle mode (i.e. not actively being used) and the ExpoM-RF4 device, while walking along the roads within the polygonal zones and while travelling by public transport between the zones. The smartphones will be carried in front of the body inside a pouch, whereas the ExpoM-RF4 will be stored in a backpack with appropriate outer compartments, maintaining a minimum distance of 30 cm from the researcher's body to prevent body-shielding effects.

Drawing from the experience gained through the pre-test and pilot studies, we have identified the most critical methods for a shortened version of the protocol to be applied in other European countries. We observed negligible variance in signal strength distributions across the tested phone models. Consequently, we decided to standardize measurements using only the Samsung A22 phone. Additionally, spot measurements will focus only on the following usage scenarios: non-use, 4G native voice call, 4G file upload, and 5G file upload. Video call scenarios were removed from the protocol as they rarely exhibited EMF exposure above the detection limits of the electric field probes. Collectively, these changes significantly reduce the protocol's length, enabling broader implementation across multiple countries without compromising the utility of the data for deriving conversion functions.

## Conclusions

The implementation of the measurement protocol in pre-test and pilot studies demonstrated its suitability for deriving functions to convert continuously collected signal strength indicators with an open-access smartphone app into electric field values. The findings from these studies underscore the necessity of expanding the protocol to include additional indoor and mobile measurements, enabling the capture of a broader range of signal strength values. Analysis of the pre-test and pilot studies revealed a positive log-linear relationship between far-field RF-EMF exposure and signal strength values, indicating that better signal quality correlates with higher levels of downlink RF-EMF exposure. When SSI data was aggregated by location, we found a strong correlation with far-field exposure, with estimated regression coefficients for the normalised LTE-RSSI value of 0.91 (95% CI: 0.55 - 1.28) in FR and 1.09 (95% CI: 0.96 - 1.22) in NL. Conversely, a negative log-linear trend was observed between EMF exposure from the handset and signal strength values, with estimated coefficients for the normalised LTE-RSSI value of -0.31 [95% CI: -0.46 - -0.16] for ear exposure and -0.20 [95% CI: -0.37 - -0.03] for chest exposure during file upload scenarios. This suggests that the app data could serve as a surrogate for exposure from the handset. Nevertheless, further data collection and analysis are needed to reduce uncertainty and improve the accuracy of exposure estimates.

## Ethical and consent

Ethics and consent were not required.

## Data Availability

The dataset used in this study is available at the Yoda data publication platform from Utrecht University: *Dataset for publication: Determining the relationship between mobile phone network signal strength and radiofrequency electromagnetic field exposure: protocol and pilot study to derive conversion functions. DOI:* https://doi.org/10.24416/UU01-OXUHTC This study contains the following underlying data: “Data” folder (Contains the raw and processed data used in the analysis). Scenarios_FR.csv and Scenarios_NL_phone_locID_yyyymmdd.csv Raw dataset containing the information collected using the ETAIN-scenarios app, including timestamps for each usage scenario. The naming convention for the files includes the country (“_NL_” or “_FR_”), the phone used to collect the data (“_phone_”), the location identifier (“_locID_”), and the date (“_yyyymmdd”). Etain_CELL_NL_phone_yyyymmdd_hhmmss.csv and Etain_CELL_FR_phone_yyyymmdd_hhmmss.csv Raw data from ETAIN 5G Scientist app for Netherlands and France. The naming convention for the files includes the country (“_NL_” or “_FR_”), the phone used to collect the data (“_phone_”), the date (“_yyyymmdd_”) and time (“_hhmmss”) of data collection. Expom_yyyymmdd_hhmmss.csv Raw data from ExpoM-RF4 exposimeter. The naming convention for the files includes the date (“_yyyymmdd_”) and time (“_hhmmss”) of data collection. Expom_yyyymmdd_hhmmss_corr.csv Cross-talk corrected data from ExpoM-RF4 exposimeter. The naming convention for the files includes the date (“_yyyymmdd_”) and time (“_hhmmss”) of data collection. ProbeLog1_NL_locID_yyyymmdd.csv Raw data collected with the on-body electric field probe placed at the right ear. The naming convention for the files includes the country (“_NL_”), the location identifier (“_locID_”), and the date (“_yyyymmdd”) of data collection. ProbeLog2_NL_locID_yyyymmdd.csv Raw data collected with the on-body electric field probe placed at the chest. The naming convention for the files includes the country (“_NL_”), the location identifier (“_locID_”), and the date (“_yyyymmdd”) of data collection. SCENARIOS_data.rds Processed dataset from ETAIN-scenarios app. ETAIN_CELL_data.rds Processed dataset from ETAIN 5G Scientist app. ETAIN_CELL_sc.rds Processed dataset combining ETAIN-scenarios (SCENARIOS_data.rds) and ETAIN 5G Scientist (ETAIN_CELL_data.rds) data. The dataset include app data by unique combination of location, phone, network provider, and scenario. EXPOM_data.rds Processed dataset from ExpoM-RF4 exposimeter. EXPOM_sc.rds Processed dataset combining ETAIN-scenarios (SCENARIOS_data.rds) and ExpoM-RF4 data (EXPOM_data.rds). The dataset include ExpoM-RF4 data by unique combination of location, phone, network provider, and scenario. PROBE_data.rds Processed dataset from on-body electric field probes. PROBES_sc.rds Processed dataset combining ETAIN-scenarios (SCENARIOS_data.rds) and electric field probes data (PROBE_data.rds). The dataset include probe data by unique combination of location, phone, network provider, and scenario. etain_allDL_nl.rds and etain_allDL_fr.rds Mean aggregates dataset from 5G Scientist, ExpoM-RF4, and probes for far-field analysis (Netherlands and France). etain_allUL_nl.rds Mean aggregates dataset from 5G Scientist, ExpoM-RF4, and probes for near-field analysis (Netherlands and France). “Data_codebooks” folder (Documentation of the dataset variables, including definitions, units, and data types). “Scripts” folder (RScripts used for data processing, analysis, and visualization). Data is publicly available under the Creative Commons Attribution 4.0 International License. Users are free to share, adapt, and use the dataset, provided appropriate credit is given. Yoda data publication platform from Utrecht University: *Dataset for publication: Determining the relationship between mobile phone network signal strength and radiofrequency electromagnetic field exposure: protocol and pilot study to derive conversion functions.* DOI: https://doi.org/10.24416/UU01-OXUHTC This study contains the following extended data: •   ExtendedData.pdf (tables and figures with direct link to the main text) •   STROBE_checklist.pdf (see Reporting guidelines section)

## Software availability

This study used the following open-source software:

•   QGIS for location visualisation and selection. Available at: http://qgis.org

•   R for data analysis within the RStudio environment. Available at: https://www.r-project.org/ and https://posit.co/

R scripts used for data cleaning and analysis are available on the Yoda data publication platform from Utrecht University. These scripts ensure that all data processing steps can be replicated and verified. The dataset and scripts can be accessed at:

- Dataset for publication: *Determining the relationship between mobile phone network signal strength and radiofrequency electromagnetic field exposure: protocol and pilot study to derive conversion functions.* DOI: https://doi.org/10.24416/UU01-OXUHTC

## Reporting guidelines

This study was conducted in accordance with the STROBE guidelines for observational studies. The checklist is included in the Extended Data, available on the Yoda data platform hosted by Utrecht University: *Dataset for publication: Determining the relationship between mobile phone network signal strength and radiofrequency electromagnetic field exposure: protocol and pilot study to derive conversion functions.* DOI: https://doi.org/10.24416/UU01-OXUHTC
