# Supplementary material for: Determining the relationship between mobile phone network signal strength and radiofrequency electromagnetic field exposure: protocol and pilot study to derive conversion functions
Source: Open Res Eur. 2025 Mar 31;4:206. Originally published 2024 Sep 19. [Version 2] doi: 10.12688/openreseurope.18285.2 (PMC12032521; doi:10.12688/openreseurope.18285.2)
Supplement: Peer review history

# Reviewer report: Reviewer response for version 2

**DOI:** 10.21956/openreseurope.21367.r52576

**Published:** 2025-05-08

**Authors:** Pablo Luis López Espí

**Affiliations:** 1 University of Alcalá, Alcalá de Henares, Spain

Many thanks to the authors for their response to my previous suggestions.

 I believe that at this stage the proposal can be accepted

Is the study design appropriate for the research question?

Yes

Is the rationale for, and objectives of, the study clearly described?

Yes

Are sufficient details of the methods provided to allow replication by others?

Yes

Are the datasets clearly presented in a useable and accessible format?

Yes

Reviewer Expertise:

Microwave and Antennas, EMF Measurements

I confirm that I have read this submission and believe that I have an appropriate level of expertise to confirm that it is of an acceptable scientific standard.

# Reviewer report: Reviewer response for version 2

**DOI:** 10.21956/openreseurope.21367.r52702

**Published:** 2025-05-08

**Authors:** Dirk Heberling, Christian Bornkessel

**Affiliations:** 1 RWTH Aachen University, Aachen, Germany; 2 RF and Microwave Research Group, Technical University Ilmenau, Ilmenau, Germany

The paper describes a method for determining conversion factors in order to draw conclusions about the strength of the electric field in which the phone is located from parameters read out with a software-modified cell phone (in particular RSRP, RSSI). The investigations are described in a comprehensible manner. They are very extensive; in particular, measurements were carried out at four locations in Lyon and 14 locations in the Netherlands, each with three cell phones, several network operators and ten different usage scenarios. A common conversion factor for far-field exposure was aggregated from the measurements by averaging over all frequencies, cell phones, locations and network operators; values for ear and chest exposure were derived for near-field exposure from the user's own cell phone.

 Our studies, cited as [20] in the document, had shown, that direct calibration measurements of the mobile phones in comparison to frequency and code-selective measuring devices for electromagnetic fields, e.g. the Narda SRM-3006, for each mobile and frequency band separately, result in much more accurate conversion factors. Beside of this our studies showed a very rough measurement discretisation of the mobile and a quite low upper dynamic rage. So strong field strength, which are of great interest, cannot be evaluated using the RSSI, because the maximum range is reached already. In our view an alternative for LTE, 5G and UMTS can be to measure the field strengths code-selectively at a measurement location by the load-independent signalling channels (RS0...RS3 for LTE, SSS for 5G, CPICH for UMTS) and link them to the corresponding SSI values of the cell phone (LTE_X_rsrp, NR_X_ssRSRP and WCDMA_X_rsrp). Compared to the procedure described in the paper, this would have the advantage that a correlation is made to the field strength that is specifically generated by these signalling signals and not to the field strength in the entire frequency band of the respective radio service, which also includes other operators. If a link is to be made with RSSI instead of RSRP, a channel power measurement is carried out in the downlink frequency band of the respective operator for the respective technology instead of the code-selective measurement. This results in conversion factors that are frequency-specific and mobile phone-specific. It is expected that these frequency-specific factors in particular will lead to a more accurate exposure assessment than factors that are averaged over all frequencies and cell phone types. This is because, strictly speaking, the conversion factor determined here by averaging over all frequencies can only be applied to networks that contain a similar frequency-related composition of the signal. If the proportional frequency composition changes, the conversion factor becomes increasingly inaccurate. It should be possible to take the frequency-specific factors into account in the app, as the frequency of the serving cell and the neighbouring cells is also included in each case.

 Having on mind that 5G will be the future network standard, we consider the assessment of 5G MaMIMO as very important, which only plays a subordinate role in the current study due to the lack of availability of corresponding base stations: In our experience the read-out power value of SSS in the SSB signalling channel is not a suitable predictor for the exposure present, at least if the station has an appreciable utilization. This is due to the fact that with 5G, the share of signalling in the total exposure at full capacity is only about 1% in terms of power. In addition, to our knowledge and according to the information of some system technology manufacturers, the traffic beam designed for data transmission may have a significantly higher gain than the broadcast beam for signalling. A more suitable SSI would have to be found here, which may only be transmitted during an active connection. In this context, the question also arises as to why file uploads were taken into account in the usage scenarios, but not data-intensive file downloads, which can lead to a relevant utilization of the base station when downloading movies, for example.

 In addition to these general comments, the following detailed questions arise:

 In the introduction the authors mention, that “…UL exposure is self-induced, more localized, and originates from near-field sources situated within the Fraunhofer limit of the human body’s position…” To our experience this is even more closed and should be considered as the zone of reactive nearfield.

 The authors explained that they use the number of deployed antennas as a measure for the network density and used the network density as a measure for the network load. To our experience this is not very strong correlated as a base station on a rural environment might have a much higher load because it is the only one and many antennas are often not an indicator for a high network density.

 In Figure 1 the starting frequency of the Expom-RF4 is give as 50Hz. To our knowledge it starts at 50 MHz.

 In the chapter methods subchapter measurement tools the authors write"...refers to the specific cell within the network currently providing telecommunication services to the mobile phone."

 How are signals from specific cell identified and used? How are more advanced technologies like carrier aggregation identified and handled?

 There is contradicting information about the frequency range of the on-body field sensors.  Figure 1 mention 100Hz – 5.800MHz and on page 7 100 – 5.800MHz is given, which we would read as 100MHz – 5.800MHz.

 For Figure 2 we recommend to use the same sequence of user scenarios for both graphs. It seemed that especially in France the traffic load was not very high in comparison to the no-use case. Does this really describe a worth case?

 In our opinion the far-field conversion factor, as explained on page 12, should not depend on the country scenario but purely on the sensor type, in detail the frequency dependent antenna factor and calibration of the phone.

Is the study design appropriate for the research question?

Partly

Is the rationale for, and objectives of, the study clearly described?

Yes

Are sufficient details of the methods provided to allow replication by others?

Yes

Are the datasets clearly presented in a useable and accessible format?

Yes

Reviewer Expertise:

Electromagnetic fields, antennas antenna measurement, eletromagnetic exposure, radar technology

We confirm that we have read this submission and believe that we have an appropriate level of expertise to confirm that it is of an acceptable scientific standard, however we have significant reservations, as outlined above.

# Reviewer report: Reviewer response for version 2

**DOI:** 10.21956/openreseurope.21367.r52701

**Published:** 2025-04-25

**Authors:** Leonidas Gavrilas

**Affiliations:** 1 University of Ioannina, Ioannina, Greece

**Full Review Report**

**Summary of the Article**

 The article by Sandoval-Diez et al., titled “Determining the relationship between mobile phone network signal strength and radiofrequency electromagnetic field exposure: protocol and pilot study to derive conversion functions”, describes a comprehensive study protocol and initial pilot studies aimed at developing reliable formulas for converting mobile phone network signal strength indicators (SSIs) into estimates of radiofrequency electromagnetic field (RF-EMF) exposure. The researchers developed an open-access smartphone application, **"**ETAIN 5G-Scientist." They systematically evaluated its performance in estimating RF-EMF exposure by comparing app-generated data with precise measurements obtained from a professional exposimeter (ExpoM-RF4) and wearable on-body electric field probes.

 The study occurred in various locations in France and the Netherlands and tested different smartphones, mobile network providers, and usage scenarios (e.g., voice calls, video streaming, data uploads). The authors found a log-linear relationship between LTE signal strength indicators and far-field RF-EMF exposure. In contrast, near-field handset-related exposure showed a negative correlation with SSIs. The findings suggest considerable potential for using smartphone apps for large-scale and citizen-involved scientific assessments of RF-EMF exposure. However, the authors note that further data collection and analysis are necessary to address uncertainties and enhance accuracy.

**Evaluation and Specific Comments**

 Below are the evaluations addressing specific aspects of the manuscript:

**1. Clarity of Rationale and Objectives**

**Answer:** Yes, clearly described.

 The rationale and objectives of the study were very clearly defined. The authors effectively contextualized their work within the growing complexity of modern mobile communication technologies and the current methodological limitations of measuring RF-EMF exposure. They convincingly explained their choice of developing smartphone-based solutions as a feasible and scalable alternative. Objectives were explicitly outlined, clearly stating the goal to establish relationships between network SSIs and RF-EMF exposure, thus facilitating future large-scale epidemiological studies.

**2. Appropriateness of Study Design**

**Answer:** Yes, appropriate.

 The study design was well-structured and appropriate for addressing the research question. Standardized usage scenarios and professional RF-EMF measurement tools alongside the smartphone app allowed rigorous and reliable comparisons. The authors carefully selected diverse environments (urban vs. rural, indoor vs. outdoor) and multiple phone models and network providers, ensuring representativeness and enhancing generalizability.

**3. Replicability of Methods**

**Answer:** Yes, fully replicable.

 The methods were comprehensively detailed, sufficiently allowing replication. Explicit descriptions of location selection criteria, measurement setups, device specifications, data collection procedures, data analysis, and aggregation were provided. Extensive supplemental information further facilitated complete methodological transparency.

**4. Accessibility and Presentation of Datasets**

**Answer:** Yes.

 The data presented were precise, well-summarized, and effectively supported the authors' conclusions through descriptive statistics, regression analyses, and graphical visualizations.

**Paper Structure**

**1. Introduction and Objectives**

 The introduction of the paper effectively contextualizes the significance of the research, clearly explaining how rapidly evolving mobile communication technologies create challenges in accurately assessing radiofrequency electromagnetic field (RF-EMF) exposure. It introduces relevant concepts such as uplink and downlink exposure, emphasizing the need for innovative assessment methods due to technological advancements and existing limitations of conventional instruments. The authors highlight the advantages of leveraging smartphone technology for scalable, population-level exposure monitoring through citizen science approaches. However, the introduction could be improved by explicitly identifying specific gaps in previous research or indicating how current smartphone-based apps fall short of adequately estimating RF-EMF exposure. Clarifying the exact novel contributions of the ETAIN 5G-Scientist app compared to existing applications would strengthen the rationale. Overall, the objectives are explicitly stated, well-aligned with the research context, and sufficiently precise.

**2. Methods – Spot Measurement Protocol**

 The methodological section is thorough and provides extensive details required for replication, including the criteria for selecting measurement locations, the setup of devices used (ExpoM-RF4 exposimeter, smartphones, on-body electric field probes), and clearly defined usage scenarios. The authors adequately justified their choices, detailing the differences in microenvironments (urban vs. rural, indoor vs. outdoor) and emphasizing standardization of usage scenarios. Nonetheless, the description could benefit from explicitly addressing practical challenges encountered during data collection, such as equipment malfunctions or environmental interference. Additionally, the synchronization approach between devices could be more clearly described, specifying precisely how temporal alignment was achieved and validated, to enhance replicability further.

**3. Pre-test and Pilot Studies – Data Analysis and Modelling**

 The analytical approach adopted is well-suited to the research question, with comprehensive explanations on normalization methods, regression modelling, and data aggregation into two-minute intervals. The choice of log-linear regression for establishing conversion functions is appropriate and justified.

**4. Results – Descriptive statistics, Far-field and Near-field Conversion Functions**

 The results section presents descriptive statistics supported by compelling visualizations. It provides valuable insights into signal strength indicators and RF-EMF exposure relationships, particularly the observed positive log-linear relationships for far-field exposure and negative log-linear relationships for near-field handset-related exposure. The regression analyses are reported, with explicit statistical parameters to evaluate model performance. However, the authors could improve this section by explicitly addressing potential outliers or extreme values in the data, detailing any sensitivity analyses conducted to ensure the robustness of the results. Moreover, explicitly acknowledging limitations arising from the relatively small sample sizes in some scenarios (particularly near-field measurements) would provide additional transparency and help contextualize findings more realistically.

**5. Discussion and Study Insights – Protocol Changes**

 The discussion section provides valuable insights into the implications of the findings and identifies uncertainties inherent in the study. It outlines proposed protocol modifications, including expanded measurements in more diverse indoor environments and increased focus on low-frequency bands. This reflective approach significantly contributes to the manuscript's strength, offering clear future directions and practical improvements. However, the authors could deepen the discussion by explicitly addressing potential practical consequences of these protocol adjustments in terms of feasibility, costs, and resource implications. Furthermore, acknowledging explicitly any biases that might result from the methodological choices (e.g., selection of smartphone models, differences in infrastructure across locations) would enhance the discussion's comprehensiveness.

**6. Conclusions**

 The conclusions are concise and effectively summarize the main findings, highlighting the potential of the smartphone app approach for large-scale RF-EMF exposure assessments. The authors correctly note that additional extensive measurements are necessary to refine and validate their conversion functions, offering a realistic appraisal of current limitations and future research requirements.

**Final Conclusion**

 The article is overall scientifically rigorous, clearly written, and methodologically sound. It contributes to RF-EMF exposure assessment methodologies, particularly by leveraging citizen science and ubiquitous smartphone technology.

Is the study design appropriate for the research question?

Yes

Is the rationale for, and objectives of, the study clearly described?

Yes

Are sufficient details of the methods provided to allow replication by others?

Yes

Are the datasets clearly presented in a useable and accessible format?

Yes

Reviewer Expertise:

Electromagnetic radiation perception and misconceptions, radiofrequency electromagnetic exposure, wireless communication systems, transmission and reception of RF signals, health-related attitudes toward EMR, scientific literacy in EMR. STEM education, educational robotics, ICT integration in learning environments, early childhood science education, environmental education.

I confirm that I have read this submission and believe that I have an appropriate level of expertise to confirm that it is of an acceptable scientific standard.

# Reviewer report: Reviewer response for version 2

**DOI:** 10.21956/openreseurope.21367.r52577

**Published:** 2025-04-12

**Authors:** Rosa Orlacchio

**Affiliations:** 1 IMS laboratory, University-École Pratique des Hautes Études (EPHE),, Paris, France

I thank the authors for their reply, and I have no further comments to make.

Is the study design appropriate for the research question?

Yes

Is the rationale for, and objectives of, the study clearly described?

Yes

Are sufficient details of the methods provided to allow replication by others?

Yes

Are the datasets clearly presented in a useable and accessible format?

Yes

Reviewer Expertise:

Therapeutic bioelectromagnetics, experimental electromagnetic dosimetry

I confirm that I have read this submission and believe that I have an appropriate level of expertise to confirm that it is of an acceptable scientific standard.

# Reviewer report: Reviewer response for version 1

**DOI:** 10.21956/openreseurope.19762.r44498

**Published:** 2024-11-04

**Authors:** Pablo Luis López Espí

**Affiliations:** 1 University of Alcalá, Alcalá de Henares, Spain

The proposal presents an interesting analysis of signal levels received with a mobile phone compared to existing field levels measured with a personal exposure meter. Two measurement locations are analysed in the manuscript: one in France and one in the Netherlands. Different scenarios are also analysed as a function of usage and technology. In the proposal, two essentially different quantities are being compared: The measured power of the control signals of the different technologies (RSCP, RSSI...) on a smartphone and the electric field strength of the exposure measured with an exposure meter.

 Overall, the proposal is well presented and of scientific interest. However, some issues should be more clearly explained:

 - The authors indicate that they do not know if any study has been carried out on this aspect, however, there is a recent reference (1) where the conversion between the two terms and some possible error factors are explained.

 - Taking into account this conversion, if we assume linearity of both receivers in their full measurement range, the relationship presented in Eq1 as a linear regression should only remain as an offset term (Beta1=1) and perhaps this explains the poor values of R^2 in the fits in figures 5, 6 and 8 and, on the contrary, the goodness of fit in figure 7.

 - It should also be explained in more detail how all the contributions detected by the smartphone have been agregated: sum of the dBm powers of the different signals of the same technology (e.g. sum of RSSI of the different BTS or operators to compare with the exposure meter value) or any other chosen method. If only the main operator is considered, this can lead to significant errors.

 - The authors propose a 2-minute data aggregation. This raises several questions: How often have the signal levels been measured that give rise to this aggregation, why has this interval been chosen rather than the usual 6 minutes for averaging exposure data, how have the aggregated mobile and exposure meter measurements been synchronised, and is there the same amount of raw data in both sets of measurements?

 - It is also worth asking about the aggregation of data whether the average is a representative value due to the temporal variability of the values detected by the mobile due to signal losses, multipaths, etcetera. Should we discard the lowest values in the same vay of that we discard losses in the smartphone?

 - Another question to analyse is that the probe for on-body measurements has a lower detection limit of 2 V/m (page 9),  which is a value far above the values measured with the exposure meter, so that the validity of the results obtained could be questioned. Could it be a typo?

Is the study design appropriate for the research question?

Yes

Is the rationale for, and objectives of, the study clearly described?

Yes

Are sufficient details of the methods provided to allow replication by others?

Yes

Are the datasets clearly presented in a useable and accessible format?

Yes

Reviewer Expertise:

Microwave and Antennas, EMF Measurements

I confirm that I have read this submission and believe that I have an appropriate level of expertise to confirm that it is of an acceptable scientific standard, however I have significant reservations, as outlined above.

## References

[1] : Smartphone-Based Methodology Applied to Electromagnetic Field Exposure Assessment. *Sensors (Basel)* .2024;24(11) : 10.3390/s24113561 10.3390/s24113561 38894351 PMC11175359

# Response

**Published:** 2025-03-24

**Authors:** Nekane Sandoval-Diez

**Affiliations:** Epidemiology and Public Health, Schweizerisches Tropen- und Public Health-Institut, Allschwil, Basel-Landschaft, Switzerland

**Review 2 by** **Pablo Luis López Espí (University of Alcalá, Alcalá de Henares, Spain)** ***Comment***: The proposal presents an interesting analysis of signal levels received with a mobile phone compared to existing field levels measured with a personal exposure meter. Two measurement locations are analysed in the manuscript: one in France and one in the Netherlands. Different scenarios are also analysed as a function of usage and technology. In the proposal, two essentially different quantities are being compared: The measured power of the control signals of the different technologies (RSCP, RSSI...) on a smartphone and the electric field strength of the exposure measured with an exposure meter. Overall, the proposal is well presented and of scientific interest. However, some issues should be more clearly explained.

***Answer***: We sincerely appreciate the reviewer's insightful comments and constructive feedback, which have helped us improve our manuscript. Below, we provide detailed responses to each point raised.

***Comment***: The authors indicate that they do not know if any study has been carried out on this aspect, however, there is a recent reference (1) where the conversion between the two terms and some possible error factors are explained.

***Answer***: Thank you for pointing out this relevant publication. We have now included it in the introduction/discussion and referenced it accordingly.

***Comment***: Taking into account this conversion, if we assume linearity of both receivers in their full measurement range, the relationship presented in Eq1 as a linear regression should only remain as an offset term (Beta1=1) and perhaps this explains the poor values of R^2 in the fits in figures 5, 6 and 8 and, on the contrary, the goodness of fit in figure 7.

***Answer***: We agree that the performance differences between Figures 5, 6, and 8 versus Figure 7 highlight the impact of various sources of uncertainty of each model, leading to β1<1.  These uncertainties are captured by the error term εᵢ in Equation 1, and we have expanded our discussion to acknowledge and elaborate on these sources of uncertainty. Our goal was to empirically assess the relationship between SSI and exposure in real-world conditions, recognizing that a perfect match (β1 = 1) is unlikely due to inherent measurement uncertainties, environmental variability, and device-specific factors.

***Comment: *** It should also be explained in more detail how all the contributions detected by the smartphone have been aggregated: sum of the dBm powers of the different signals of the same technology (e.g. sum of RSSI of the different BTS or operators to compare with the exposure meter value) or any other chosen method. If only the main operator is considered, this can lead to significant errors.

***Answer:*** Thank you for this observation. We have now expanded the explanation of our aggregation method for the map conversion function, clarifying how signal contributions were combined.

***Comment: *** The authors propose a 2-minute data aggregation. This raises several questions: How often have the signal levels been measured that give rise to this aggregation, why has this interval been chosen rather than the usual 6 minutes for averaging exposure data, how have the aggregated mobile and exposure meter measurements been synchronised, and is there the same amount of raw data in both sets of measurements?

***Answer: *** Thank you for your observation. We have added more details on this aspect in the methods section. We chose a 2-minute data aggregation period because each usage scenario was designed to last for 2 minutes. This duration was selected to ensure the feasibility and practical applicability of the protocol. Since these were spot measurements, extending the measurement duration beyond 2 minutes would not have provided substantially more information. Furthermore, we did not apply the typical 6-minute averaging time or other guideline-based averaging periods, as the aim of the app was not to assess compliance with exposure guidelines but rather to characterize exposure under specific usage conditions. Regarding measurement frequency, SSI data from the app was recorded every second, while ExpoM-RF4 measurements were taken every 7 seconds. Both datasets included timestamps, which allowed us to synchronise them with each other and with the timestamps from the ETAIN-scenarios bespoke app used to log usage scenarios. This temporal alignment ensured consistency across the mobile app and exposimeter data for aggregation and analysis.

***Comment: *** It is also worth asking about the aggregation of data whether the average is a representative value due to the temporal variability of the values detected by the mobile due to signal losses, multipath, etcetera. Should we discard the lowest values in the same way of that we discard losses in the smartphone?

***Answer:*** We acknowledge that SSI values are subject to temporal variability due to signal losses, multipath effects, and other environmental factors. We chose to use the arithmetic mean SSI as it provides a stable representation of the signal strength experienced at a given location when connected to a certain cell. Additionally, the measurement devices have different sampling rates (ExpoM-RF4 measures exposure every 7 seconds, while the app logs SSI data every second). While percentile-based aggregation could be considered, it may not be as robust in this context due to the mismatch in sampling frequency. Using percentiles instead of averages could introduce additional uncertainty by comparing distributions with different granularities. To further assess this, we performed a sensitivity analysis using the 95 ^th^ percentile aggregation instead of the average. The results did not materially change (see figure below for technical conversion function fitted using 95 ^th^ percentile aggregates).

Figure 1- 18285 (OREU).docx 

 Figure 1. Regression analysis of active-band downlink field strength vs normalised LTE-RSSI from serving cell using 95 ^th^ percentile values. *The dots represent measurement aggregates (two-minute 95 ^th^ percentile aggregates subdivided by registered cells) colour- and shape-coded according to frequency band. The red lines show the regression fit, and the grey areas represent 95% confidence bounds.*  

***Comment: *** Another question to analyze is that the probe for on-body measurements has a lower detection limit of 2 V/m (page 9), which is a value far above the values measured with the exposure meter, so that the validity of the results obtained could be questioned. Could it be a typo?

***Answer:***  Unfortunately, the lower detection limit of the field probes is indeed 2 V/m. Unlike the ExpoM-RF4, the probes used in this study are broadband and were specifically designed for on-body measurements, focusing on exposure from the handset. Exposure from near-field sources was expected to be orders of magnitude higher than far-field exposure when the phone is used close to the body. We acknowledge that the 2 V/m detection limit is a technical limitation of the probes. As noted in the paper, when limiting the analysis to measurements above this threshold, only a few measurements exceeded it, and for most usage scenarios, the exposure remained below this limit. Despite this, we were still able to observe the expected negative correlation between handset exposure and signal strength in our results.

# Reviewer report: Reviewer response for version 1

**DOI:** 10.21956/openreseurope.19762.r44495

**Published:** 2024-10-24

**Authors:** Rosa Orlacchio

**Affiliations:** 1 IMS laboratory, University-École Pratique des Hautes Études (EPHE),, Paris, France

The authors outline the development and evaluation of the "5G Scientist Monitor," an open-access mobile app created within the frame of the EU project ETAIN to estimate radiofrequency electromagnetic field (RF-EMF) exposure from smartphones using different signal strength indicators (SSIs).

 The paper is very interesting and well-written. Please, find below a few comments.

Abstract.

 RSSI. If it is the R coefficient of the linear regression I would express it as R _SS _Otherwise, please define it.

Introduction.

 “Several studies have reported a robust negative correlation between SSIs and transmitted power”. It would be of interest to explain why this correlation is negative.

 It is unclear what is different about this new app, “5G Scientist Monitor,” compared to the ones you mentioned, i.e., XMobiSense, ElectroSmart, Quanta Monitor, and QualiPoc Android.

Methods.

Measurement Tools.

 What about iOS (Apple) phones?

 Figure 1. A) Three broadband E-field sensors. Aren’t they 2? Please, clarify.

 Table 2. Why only the right hand?

 Have you planned to consider exposure when using headphones?

Data analysis and modeling.

 Why normalization is done for f _0_ = 1800 MHz?

 “These aggregates were computed for each unique combination of location, phone, provider, and scenario (n=795 in NL and n=325 in FR) by taking the arithmetic mean of the app SSIs, and of the recorded ExpoM-RF4 and probe electric field strengths during the interval”. You have averaged all your data without considering the location. Nevertheless, in Figure 6 you show data also as a function of the location. Could you also show the n relatives to the different locations?

 Table 3. Check the spelling of ‘receive power’ in Implications, Technical conversion function

Results.

 Location seems to play a significant role in exposure levels when measured with the app compared to the ExpoM-RF4 measurements (Fig.6). Could you comment a bit more on that?

 Moreover, it would have been interesting to see a similar data representation in Figure 5 as the one shown in Figure 6. It would also be helpful to include a legend in Figure 6 to clarify the color coding for each location. 

 Figure 7 would be clearer with a legend explaining the name of the points used for the regression. 

 “Additionally, the results of the far-field conversion function for 5G-NR showed that NR-ssRSRP was not a significant predictor of neither active-band nor NR exposure, which was always from a TDD band (Figure S10 in Extended Data28)”. Could you explain why?

 Figure 8. Please, provide more information about it in the caption. Please, justify the choice of 4G, native call, WAvoice, etc.

Discussion.

 “Our study provides initial findings regarding the complex relationship between RF-EMF exposure and SSIs within the framework of 5G-NR technology.”. However, If I have well understood, most of the data that you show are relative to 4G. Could you please comment on that?

Is the study design appropriate for the research question?

Yes

Is the rationale for, and objectives of, the study clearly described?

Yes

Are sufficient details of the methods provided to allow replication by others?

Yes

Are the datasets clearly presented in a useable and accessible format?

Yes

Reviewer Expertise:

Therapeutic bioelectromagnetics, experimental electromagnetic dosimetry

I confirm that I have read this submission and believe that I have an appropriate level of expertise to confirm that it is of an acceptable scientific standard, however I have significant reservations, as outlined above.

# Response

**Published:** 2025-03-27

**Authors:** Nekane Sandoval-Diez

**Affiliations:** Epidemiology and Public Health, Schweizerisches Tropen- und Public Health-Institut, Allschwil, Basel-Landschaft, Switzerland

***Comment*** : The authors outline the development and evaluation of the "5G Scientist Monitor," an open-access mobile app created within the frame of the EU project ETAIN to estimate radiofrequency electromagnetic field (RF-EMF) exposure from smartphones using different signal strength indicators (SSIs). The paper is very interesting and well-written. Please, find below a few comments. ***Answer: *** The authors would like to thank the reviewer for taking the time to read our manuscript and providing insightful comments and constructive feedback. Below, we address each point raised:

- Abstract.
  -
***Comment*** : RSSI. If it is the R coefficient of the linear regression I would express it as RSS Otherwise, please define it. ***Answer*** : Thank you for the suggestion. RSSI refers to the Received Signal Strength Indicator, and the acronym has now been defined in the abstract.

- Introduction
  - ***Comment: *** “Several studies have reported a robust negative correlation between SSIs and transmitted power”. It would be of interest to explain why this correlation is negative. ***Answer: *** We appreciate the comment and have added an explanation clarifying why this correlation is negative.

-
  - ***Comment: *** It is unclear what is different about this new app, “5G Scientist Monitor,” compared to the ones you mentioned, i.e., XMobiSense, ElectroSmart, Quanta Monitor, and QualiPoc Android. ***Answer*** : Thanks for the observation, we have added a sentence to make this clearer. The ETAIN 5G Scientist app, like the other mentioned apps, collects SSI data. However, it stands out by adopting a citizen science approach, involving both citizens and experts in its design to better address public needs and enhance engagement. Additionally, the ETAIN 5G Scientist app implements a reproducible and documented framework for estimating RF-EMF exposure from app-collected data, which distinguishes it from other apps that often provide limited details about their calculation processes.

- Methods. Measurement tools
  - ***Comment: *** What about iOS (Apple) phones? ***Answer*** : Developing the app for iOS posed significant challenges due to Apple's stricter access policies regarding internal phone parameters. These limitations made it complicated to integrate the app into the Apple ecosystem. As a result, the app is only available on Android.

-
  - ***Comment: *** Figure 1. A) Three broadband E-field sensors. Aren’t they 2? Please, clarify. ***Answer:  *** Thank you for spotting the typo; it has been corrected in the figure.

-
  - ***Comment: *** Table 2. Why only the right hand? ***Answer*** : The right hand was specified to standardize the measurement protocol and minimize inter-operator variability.

-
  - ***Comment: *** Have you planned to consider exposure when using headphones? ***Answer*** : Our focus in this study was on RF-EMF exposure from mobile phone use and mobile telecommunication technologies. While we did not include exposure from peripherals or other wireless technologies, we recognize their relevance and consider it a topic for future research.   

- Data analysis and modelling
  - ***Comment: *** Why normalization is done for f0 = 1800 MHz? ***Answer*** : Normalisation was performed for f0 = 1800 MHz it is centrally located within the frequency bands used in mobile networks. This choice has now been clarified in the text.

-
  - ***Comment: *** These aggregates were computed for each unique combination of location, phone, provider, and scenario (n=795 in NL and n=325 in FR) by taking the arithmetic mean of the app SSIs, and of the recorded ExpoM-RF4 and probe electric field strengths during the interval”. You have averaged all your data without considering the location. Nevertheless, in Figure 6 you show data also as a function of the location. Could you also show the n relatives to the different locations? ***Answer*** : Thank you for the suggestion. We have added the sample sizes (n) for the locations and expanded the explanation of the aggregation process used for the map conversion function shown in Figure 6.

-
  - ***Comment: *** Table 3. Check the spelling of ‘receive power’ in Implications, Technical conversion function. ***Answer*** : Thank you for noting the typo. The typo has been corrected in the table.

- Results
  - ***Comment: *** Location seems to play a significant role in exposure levels when measured with the app compared to the ExpoM-RF4 measurements (Fig.6). Could you comment a bit more on that? ***Answer*** : Thank you for your comment, we have elaborated more on this in the results section. The ExpoM-RF4 measures the combined exposure from all providers, while the app captures provider-specific signals. Geographic variability in base station density and provider distribution accounts for the observed differences.  

-
  - ***Comment: *** Moreover, it would have been interesting to see a similar data representation in Figure 5 as the one shown in Figure 6. It would also be helpful to include a legend in Figure 6 to clarify the colour coding for each location. ***Answer*** : Thank you for your suggestion. We believe that adding a colour code for each location in Figure 5 would not enhance the interpretation of the results. Figure 5 presents band-specific exposure levels correlated with the SSI from the serving cell. In this context, location primarily influences the frequency band used for communication, as lower frequencies are typically employed in areas with lower network coverage. Therefore, we opted to use colour codes to represent the frequency bands in Figure 5.

-
  - ***Comment: *** Figure 7 would be clearer with a legend explaining the name of the points used for the regression. ***Answer: *** To improve clarity, we opted to change Figure 7 to include a colour code representing the level of urbanisation and type of microenvironment for each location.
-
  - ***Comment: *** “Additionally, the results of the far-field conversion function for 5G-NR showed that NR-ssRSRP was not a significant predictor of neither active-band nor NR exposure, which was always from a TDD band (Figure S10 in Extended Data28)”. Could you explain why? ***Answer:*** We elaborated an explanation for this in the discussion section.

-
  - ***Comment: *** Figure 8. Please, provide more information about it in the caption. Please, justify the choice of 4G, native call, WAvoice, etc. ***Answer:*** We have added a detailed explanation of the reasons for stratification in the methods section and have expanded the caption of Figure 8 to provide further context.

- Discussion
  - ***Comment: *** “Our study provides initial findings regarding the complex relationship between RF-EMF exposure and SSIs within the framework of 5G-NR technology.”. However, If I have well understood, most of the data that you show are relative to 4G. Could you please comment on that? ***Answer:*** Yes, most of the data in our study is based on 4G-LTE technology, as 5G-NR had not yet been deployed in the Netherlands at the time of data collection. However, we conducted a preliminary analysis of the available 5G-NR data from Lyon (F) to identify opportunities for future research.
